# Comparison of the Efficacy of Longer versus Shorter Pulsed High Dose Dapsone Combination Therapy in the Treatment of Chronic Lyme Disease/Post Treatment Lyme Disease Syndrome with Bartonellosis and Associated Coinfections

**DOI:** 10.3390/microorganisms11092301

**Published:** 2023-09-12

**Authors:** Richard I. Horowitz, John Fallon, Phyllis R. Freeman

**Affiliations:** 1 Lyme and Tick-Borne Diseases Working Group, New York State Department of Health, Albany, NY 12224, USA; 2 Hudson Valley Healing Arts Center, Hyde Park, NY 12538, USA; johnf@hvhac.com (J.F.); research@hvhac.com (P.R.F.)

**Keywords:** Lyme disease, post-treatment Lyme disease syndrome (PTLDS), dapsone combination therapy (DDSCT), double-dose dapsone combination therapy (DDDCT), high-dose dapsone combination therapy (HDDCT), babesiosis, bartonellosis, florescent in situ hybridization (FISH), persistent infection

## Abstract

Twenty-five patients with relapsing and remitting Borreliosis, Babesiosis, and bartonellosis despite extended anti-infective therapy were prescribed double-dose dapsone combination therapy (DDDCT), followed by one or several courses of High Dose Dapsone Combination Therapy (HDDCT). A retrospective chart review of these 25 patients undergoing DDDCT therapy and HDDCT demonstrated that 100% improved their tick-borne symptoms, and patients completing 6–7 day pulses of HDDCT had superior levels of improvement versus 4-day pulses if *Bartonella* was present. At the completion of treatment, 7/23 (30.5%) who completed 8 weeks of DDDCT followed by a 5–7 day pulse of HDDCT remained in remission for 3–9 months, and 3/23 patients (13%) who recently finished treatment were 1 ½ months in full remission. In conclusion, DDDCT followed by 6–7 day pulses of HDDCT could represent a novel, effective anti-infective strategy in chronic Lyme disease/Post Treatment Lyme Disease Syndrome (PTLDS) and associated co-infections, including *Bartonella*, especially in individuals who have failed standard antibiotic protocols.

## 1. Introduction

Lyme borreliosis (LB), due to an infection with *Borrelia burgdorferi* (Bb), is a rapidly growing healthcare problem across the globe. In 2022, the United States (US) had an estimated 476,000 new diagnosed cases of Lyme disease (LD) [1] with an estimated global Bb seroprevalence of 14.5% [2]. In a large European cohort, approximately 27% of those individuals exposed to Lyme borreliosis will go on to develop Post Treatment Lyme Disease Syndrome (PTLDS) despite treatment [3], and in a 2013 study of United States (US) patients, 35% of participants were diagnosed as having PTLDS [4]. At 6 months, 36% of those patients reported new-onset fatigue, 20% widespread pain, and 45% neurocognitive difficulties, significantly adversely affecting the quality of their lives [4]. Other systematic reviews of long-term sequelae in confirmed Lyme disease cases demonstrated that PTLDS may result in impaired quality of life, with patients complaining of persistent fatigue, musculoskeletal pain, paresthesias, insomnia, depression, poor appetite, and difficulties with concentration [5]. The cumulative prevalence of PLTDS in the United States in 2020 was estimated to be 2 million individuals and was continuing to increase [6], while in Europe, varying case definitions, data collection methods, and different study designs have impeded understanding the Europe-wide burden of LB [7]. It is likely to be significant since, among countries with surveillance data, 43.2% of the population live in a high LB incidence region [7]. The economic burden and aggregate cost of diagnosed Lyme disease are estimated to be nearly $1 billion dollars annually in the US in high-incidence areas, not including suspected, undiagnosed, or nonacute cases [8]. In Europe, in Belgium, a high-incidence country, estimated direct and indirect costs in 2023 were €19.4 and 151.5 million Euros, respectively, where disability benefits were associated with higher direct and out-of-pocket costs [9]. As global increases in temperature due to climate change amplify the number of vector-borne diseases worldwide, increasing numbers of ticks, mosquitos, and flea populations [10], a rising economic burden and diminished quality of life due to Lyme Borreliosis, PTLDS, mosquito-borne illness (i.e., malaria, West Nile, Zika, Chikungunya), and flea-borne illness including bartonellosis are expected to simultaneously rise [11,12,13]. Therefore, there is an urgency to find effective diagnostic and treatment protocols for chronic LD, PTLDS, and associated co-infections such as *Babesia* and *Bartonella*, which are causing significant disability and straining essential healthcare resources [14].

The etiology of chronic LD/PTLDS and associated symptomatology is a highly debated topic [15,16]. Similarly, the role of co-infections in chronic LD/PTLDS has been disputed [17], although some scientific studies have documented an exacerbation of Lyme disease symptoms with tick-borne co-infections such as *Babesia* [18] or *Bartonella* [19]. Recent scientific discoveries have documented that *Borrelia* are capable of establishing a chronic persistent infection in both animal models (mice, dogs) [20,21], non-human primates [22] and humans [23,24] despite treatment with a variety of antibiotics; similarly, *Babesia* is capable of establishing persistent infections in animal models (mice, dogs, cattle) [25,26,27], and humans [28], despite anti-infective treatment [28], as is *Bartonella*, a vector-borne, facultative, intracellular stealth pathogen [29,30]. In some studies, co-infection with *Babesia* and *Borrelia* has been shown to increase the number and severity of symptoms and result in a longer duration of illness [31,32,33,34], as has infection with *Borrelia* and *Bartonella* [19,35,36].

The most frequent scientific hypotheses to explain chronic persistent symptomatology in those suffering from CLD/PTLDS involve multiple mechanisms, including not only co-infections [37,38], but also immune evasion [39,40] with complement evasion and resistance [41], a persistent excessive, dysregulated pro-inflammatory immune response during the post-infectious period [42] persistent antigenic debris including peptidoglycans [43], altered neural networks with central sensitization [15], continuing infection with *Borrelia* [44,45,46,47,48] and/or a combination of the above [49]. Horowitz has identified a precision medicine model that has identified up to 16 factors likely responsible for chronic persistent symptomatology in patients suffering from CLD/PTLDS, referred to as MSIDS (Multiple Systemic Infectious Disease Syndrome) [50]. These include multiple overlapping sources of inflammation (borreliosis and chronic co-infections including *Bartonella*, leaky gut with dysregulation of gut microbiota, multiple food sensitivities with or without mast cell activation, environmental toxicity [heavy metals, mold], vitamin and mineral deficiencies, and sleep disorders) [51,52,53,54,55,56,57,58,59,60,61] with downstream effects of inflammation, including mitochondrial dysfunction [62], hormonal dysregulation [63,64], Postural Orthostatic Tachycardia Syndrome (POTS) with dysautonomia [65], autoimmunity [42,66], neuropsychiatric symptoms [67], liver dysfunction [68,69] and resistant pain syndromes [70]. Patients oftentimes have multiple overlapping etiologies responsible for their chronic fatiguing, musculoskeletal, and neuropsychiatric symptoms [71].

In the past several years, researchers at Johns Hopkins University, the University of New Haven, Stanford University, and Northeastern University have identified stationary, persister, and biofilm forms of *Borrelia burgdorferi* as a potential cause of persistent infection and ongoing inflammation that could account at least in part for continued symptoms in CLD/PTLDS [72,73,74,75]. These stationary, persister forms in biofilms have been found to be resistant to standard antibiotics [76] and a significant source of inflammation in the mouse model of LD, potentially causing more severe disease [77]. Previous scientific studies had hypothesized that *Borrelia* persists because of a combination of factors. These include immune evasion and changing outer surface proteins [78,79], resisting clearance by modulating the activity of immune cells involved in both the innate and adaptive responses [80] with reduced B and T cell synchronicity [81], persistence in privileged niches such as the intracellular compartment in endothelial cells [82], macrophages [83], fibroblasts [46,84], and neuronal/glial cells [47], changing morphological forms in varying environments [85,86], resulting in atypical cystic, rolled, and granular forms of Bb [87], as well as round bodies [88]. The scientific discovery of quorum sensing by *Borrelia* [89] and the formation of biofilms and persister forms of Bb [90] may account, however, for the intractability of disease, especially Lyme neuroborreliosis (LNB) [91], as biofilms have been found in chronic diseases that resist host immune responses and antibiotic treatment [92,93].

Two repurposed medications have been published to have effects on the persister/biofilm forms of Bb [73,75] and associated co-infections, *Babesia* and *Bartonella*: disulfiram (DSF) and dapsone, diaminodiphenylsulphone (DDS) [94,95]. In an initial clinical report on disulfiram, three patients with chronic relapsing neurological Lyme disease and Babesiosis who required open-ended antimicrobial therapy to control their symptoms, including intravenous (IV) therapy, discontinued treatment and remained in remission for 6–23 months following a 6-week to 5-month course of disulfiram [96]. One patient had evidence of a positive Lyme polymerase chain reaction (PCR) in the blood 6 months post-therapy, with relapsing symptoms requiring retreatment [96]. In another study of 71 Lyme disease patients on disulfiram, 36.4% went into long-term remission (operationally defined as clinically well for ≥6 months) after completing 1–2 courses of high-dose therapy for 6 weeks to 16 months [97]. Twelve of those patients were co-infected with *Bartonella henselae*, and 83.3% reported some clinical improvement, although only two patients achieved full remission with high-dose DSF alone (16.7%), while several patients on either minocycline or azithromycin with rifampin and DSF did not achieve remission [97]. The small number of patients on concurrent antimicrobial treatment with disulfiram precluded reaching any definitive conclusions on the efficacy of DSF for Lyme and bartonellosis.

Dapsone therapy, using dapsone alone, with a tetracycline and/or rifampin, or with or without azithromycin, has also been shown to be effective against the biofilm/persister forms of Bb [95]. Although dapsone as a single drug was effective, each additional intracellular antibiotic added to dapsone in culture (doxycycline, doxycycline plus rifampin, doxycycline, rifampin plus azithromycin) increased the ability of the combination therapy to further reduce the mass and viability as well as the protective mucopolysaccharide layers of *B. burgdorferi* biofilm. The 4-drug combo of dapsone, a tetracycline, rifampin, and macrolide (azithromycin) had the most significant effect [95].

Similarly, *Bartonella henselae*, a facultative intracellular gram-negative bacteria, has been found to have stationary biofilm/persister forms [98], accounting in part for its ability to form a chronic, persistent infection [99]. Other *Bartonella* species, apart from *B. henselae*, have been published to establish long-term stealth infections in immunocompetent persons, including *B. vinsonii* subsp. *berkhoffii*, *B. bacilliformis*, and *B. quintana*, leading to a chronic, persistent infectious state [100]. Among 14 antibiotics and 25 antibiotic combinations for activity against stationary phase *B. henselae* in culture, methylene blue and rifampin were the most active agents against the biofilm *B. henselae* after 6 days of drug exposure, whereas both rifampin and methylene blue and azithromycin and methylene blue completely eradicated the biofilm form of *B. henselae* in culture after treatment for 6 days [101]. To date, only two studies have examined using rifampin, azithromycin, and methylene blue along with tetracycline, rifampin, and dapsone in the treatment of chronic Lyme, *Babesia*, and chronic bartonellosis, with variable results [102,103].

In five separate clinical studies involving more than 365 patients, dapsone combination therapy led to statistically verified improvement in relieving eight major Lyme symptoms in our patients, including sweats/chills, fatigue, joint, muscle, and/or nerve pain, headaches, insomnia, and cognition [50,51,102,103,104]. In a 6th clinical case study involving chronic LD and Behçet’s disease (a severe multisystemic autoimmune illness), a patient who had been ill for 20 years reported significant symptom improvement when pyrazinamide (PZA) was added to dapsone combination therapy (DDSCT) despite a history of failing multiple disease-modifying antirheumatic drugs (DMARDS) [105]. *Bartonella* titers turned positive in this patient after adding pyrazinamide (PZA) to other intracellular antibiotics (minocycline, rifampin, and dapsone), where Behçet’s ulcers and granulomatous skin changes resolved for the first time in decades, implicating a potential role for *Bartonella* in the autoimmune manifestations of Behçet’s [105]. 

A common clinical finding in these dapsone studies for CLD/PTLDS and associated co-infections, similar to studies using DSF, is that higher doses of dapsone showed superior efficacy in putting patients into long-term remission. Double-dose dapsone combination therapy (DDSCT) using dapsone 100 mg PO BID with a tetracycline and rifampin was superior to single-dose dapsone (100 mg QD) with a tetracycline and rifampin, leading to tick-borne symptom improvements in 98% of patients, with 45% remaining in remission for 1 year or longer [102]. In those with PTLDS and *erythema migrans* (EM) rashes, 100% improved, and 58% went into long-term remission post-DDDCT [102] if all MSIDS variables were accounted for and adequately treated. However, none of these chronically ill patients who had evidence of *Bartonella* RNA in their blood and were FISH-positive achieved long-term remission post-treatment with DDDCT. Among 11 patients with a history of *Bartonella* exposure and no evidence of active infection (biopsy negative, PCR negative, FISH negative), 5 (45%) remained in remission, and 6 (55%) improved their underlying symptomatology [102]. When higher-dose pulse dapsone therapy (200 mg PO BID) was used for 4 days in those who failed to go into remission after 8 weeks of DDDCT, the majority of patients noticed sustained improvement in eight major Lyme symptoms [103]. A total of 32% (8/25) had a resolution of all active Lyme symptoms post-treatment for 3-months or longer, even if there was evidence of prior active co-infections, including *Babesia* and *Bartonella*. Of 19 patients (76%) with a history of *Bartonella* exposure, 15/19 improved (79%), and among 8 patients with proof of active *Bartonella* infection, i.e., PCR positive, FISH positive, and/or elevated vascular endothelial growth factor (VEGF), 3/8 (38%) remained in remission, with two out of four who were *Bartonella* FISH positive going into remission (50%). The conclusion of the study was that the higher dapsone dosage, not just the treatment length, positively affected outcomes [103]. 

Methylene blue (MB) was used in both the double dose and pulsed high dose dapsone studies to help lower methemoglobin levels, a known side effect of dapsone [106], at doses ranging from 50 mg PO BID to 100 mg PO BID, while combination therapy of dapsone, methylene blue, a tetracycline, rifampin, and azithromycin would be expected, based on prior published studies by Johns Hopkins researchers, to have had a positive effect on *Bartonella* persisters [98,101]. Only a small number of chronic Lyme disease (CLD) patients co-infected with *Bartonella* and *Babesia* achieved remission after 8 weeks of DDDCT and 4 days of HDDCT. This left us with several essential research questions. We knew that in culture 6 day of treatment with rifampin and/or azithromycin plus methylene blue eliminated *Bartonella* persisters. Taking these results from “bench to bedside”, would a longer 6–7 day pulse of HDDCT with higher doses of MB result in superior clinical efficacy against the most common co-infection preventing the long-term success of our DDDCT protocol, *Bartonella*? Would higher doses of methylene blue show increased efficacy against Lyme persisters, based on its identification from an FDA drug library as having anti-persister activity against *Borrelia burgdorferi* [107], apart from dapsone’s published effect as a persister drug for Bb [108]? Would one or several 6–7 pulses of HDDCT improve remission rates and avoid the need for longer-term antibiotic therapy in those with CLD/PTLDS and associated co-infections? Would adding pyrazinamide (PZA) to DDDCT and HDDCT, or nitrofurantoin, medications shown to have some efficacy against *Bartonella*, improve long-term outcomes? Would higher doses of methylene blue as well as higher doses of folic acid improve the tolerance of DDDCT and HDDCT by reducing dapsone-induced anemia and its inhibition of the folic acid pathway while further lowering methemoglobin levels, improving tolerability [109,110]? In the present study, we evaluated 25 patients who completed a course of DDDCT, followed by either one or several 4-day or 67 day pulses of HDDCT. We compared the short-term versus long-term efficacy and tolerability of the two protocols, which varied in length, dosage of methylene blue, dosage of folic acid (using Leucovorin, i.e., folinic acid and L-methylfolate), and use of associated antibiotics, including PZA and macrodantin (Nitrofurantoin). A retrospective chart review of these patients demonstrated that, in total, 100% of patients improved their tick-borne symptoms post-HDDCT, despite some having active co-infections (*Babesia* and *Bartonella*). Among 23 patients who completed a full 8-week DDDCT protocol prior to at least one 4-day or 5–7 day HDDCT pulse, 7/23 (30.4%) went into full remission for 3-months or longer, and 3/23 (14%) went into remission for 6 weeks or longer. 

## 2. Materials and Methods

We closely examined 25 charts of adult patients who completed the DDDCT and HDDCT protocols.

A total of 19 of the 25 patients (76%) had previously completed one course of DDDCT; 2 patients (8%) completed 2 courses of DDDCT; and 2 patients (8%) completed 3 courses of DDDCT before doing one or several courses of HDDCT, which consisted of either a short pulse (4 days) or a longer pulse (5–7 days). Three patients in this chart review had previously undergone one or more treatments with disulfiram and/or DDDCT and had either failed or had an inadequate response to prior antibiotic therapy and/or had relapsed with persistent symptoms after stopping anti-infective therapy. Three patients were also unable to complete the protocol as prescribed. Two patients (8%) did not complete a full 28-day course of DDDCT prior to completing a HDDCT pulse. One was due to a severe Herxheimer flare with neuropsychiatric symptoms secondary to DSF use with DDS, and another was due to viral gastroenteritis. Another patient was unable to complete the 6-day HDDCT pulse as prescribed with 300 mg of MB due to his history of severe neuropsychiatric symptoms with psychosis, requiring a continuation of his psychiatric medicine. 

We assessed co-infection status, age, gender, length of illness, and response to treatment, i.e., self-reported improvement in Lyme symptoms, and whether there was remission, percentage improvement, or lack of response to the HDDCT protocol. Full remission was defined as the resolution of all active tick-borne symptoms for at least 3 months post-therapy. Partial remission was defined as the resolution of all active tick-borne symptoms for at least 6 weeks post-therapy since some patients had recently finished the HDDCT protocol. All 25 patients in our retrospective chart review met the criteria for a clinical diagnosis of Lyme disease supported by a physician-documented erythema migrans (EM) rash and/or positive laboratory testing, including a positive ELISA/enzyme immunoassay (and/or C6 ELISA), immunofluorescent antibody (IFA), Centers for Disease Control and Prevention (CDC) positive IgM and/or IgG Western Blot (WB), PCR, *Borrelia*-specific bands (23, 31, 34, 39, 83/93) on a WB [111,112], and/or positive ELISpot (lymphocyte transformation test (LTT)). 

All patients signed informed consent forms that outlined the proposed benefits and the potential risks of treatment. Patients volunteered to take the high-dose pulsed dapsone protocol at our medical practice based on our prior research illustrating the benefit of dapsone combination therapy in the treatment of CLD/PTLDS [51,102,103,104] and on the drug’s documented action on “persister” bacteria in biofilms [95]. None of the patients had a significant sulfa allergy or G-6-P-D deficiency, in order to minimize the possibility of allergic reactions or severe hemolytic anemia secondary to dapsone. Two patients who reported a history of a prior rash to sulfamethoxazole/trimethoprim (Bactrim DS) were given an H1/H2 blocker (cetirizine 10 mg/famotidine 10 mg) prior to starting low-dose dapsone (25 mg QOD) and had no rashes or allergic reactions during the entire course of therapy. Prior to beginning HDDCT, patients were required to have a hemoglobin greater than 12 mg/dL, no active bleeding disorders, and no contraindications or significant allergies to any of the medications or supplements. The side effects of dapsone were explained in detail, including potential rashes, Herxheimer reactions, anemia, and methemoglobinemia [109,113]. The patients were asked to obtain a baseline methemoglobin level before starting DDDCT and pulsed HDDCT to ensure that there were no significant baseline elevations due to genetic variations or other medication interactions [110]. A complete blood count (CBC) and comprehensive metabolic profile (CMP) with electrolytes, kidney, and liver function, as well as methemoglobin levels, were obtained before, during, and after therapy. Laboratory evaluations were performed during week 3, and then weekly on DDDCT (weeks 5–8) and HDDCT (week 9), as well as 3 and 8 weeks after the completion of therapy, to ensure the reversal and normalization of any laboratory abnormalities. Healthcare staff closely monitored laboratory test results during and after DDDCT and HDDCT, and an emergency phone number was provided to all patients if urgent questions arose. A baseline EKG was also required prior to starting hydroxychloroquine, azithromycin, and/or ondansetron to rule out any associated QT prolongation [114] or arrhythmias, and patients were requested to repeat an EKG once all medications that could prolong the QT interval were on board. The potential side effects of HDDCT were addressed using medication and nutritional supplements and antioxidants with anti-inflammatory effects, including glutathione, N-acetylcysteine, and alpha lipoic acid, which block NFKappaB [115], and turmeric and broccoli seed extract (sulforaphane glucosinolate), which stimulate Nrf2 [116,117]. Glutathione, nicotinamide adenine dinucleotide with hydrogen (NADH), Vitamin C, Vitamin E, and methylene blue were all used to decrease methemoglobin levels [106,118,119], along with occasional use of cimetidine to lower methemoglobin in those with a history of significant methemoglobinemia [120,121], while high dose folic acid (folinic acid, i.e., Leucovorin and L-methyl-folate) was prescribed to minimize anemia from inhibition of bacterial synthesis of dihydrofolic acid by dapsone [122]. Folinic acid supplementation has been shown in prior studies to help limit myelosuppression, gastrointestinal toxicity, nephrotoxicity, and neurotoxicity that can result from high dosages of folic acid antagonists [122]. High-dose probiotics, including *Saccharomyces boulardii*, were used to help prevent antibiotic-associated diarrhea [123,124].

In order to minimize potential drug interactions with methylene blue, including serotonin syndrome/toxicity [125], patients on psychiatric medications consulted with their prescribing physician and/or mental health professionals regarding their ability to safely taper their psychiatric medications before beginning dapsone combination therapy and methylene blue. This is especially important if the patient is on a selective serotonin reuptake inhibitor (SSRI), serotonin and norepinephrine reuptake inhibitor (SNRI), monoamine oxidase inhibitor (MAOI), and/or bupropion (Wellbutrin). There are also potential interactions between methylene blue (MB) and certain narcotics for pain, as well as herbal therapies. A drug interaction checker was therefore used before instituting MB to review potential polypharmacy interactions. Patients were given a typed MB consent form prior to instituting MB (Supplement S1) which highlighted potential drug interactions and side effects before beginning double-dose dapsone combination therapy (DDDCT) or high-dose dapsone combination therapy (HDDCT).

Dietary restrictions were also instituted, avoiding foods high in tyramine, which can potentially produce a hypertensive crisis in the presence of MAOIs and/or higher doses of methylene blue [126]. Patients were given a list of foods to avoid during the protocol, which included: aged cheese, aged chicken or beef liver, air-dried sausage and similar meats, avocados, beer, and wine (in particular, red wine), canned figs, caviar, fava beans, meat tenderizer, overripe fruit, pickled or cured meat or fish, raisins, sauerkraut, shrimp paste, sour cream, soy sauce, and yeast extracts [127]. Phenazopyridine (Pyridium) was used at a dose of 200 mg TID prn if any urinary discomfort/burning arose from the use of higher doses of methylene blue [128].

Several patients took disulfiram (DSF) in combination with HDDCT if they had previously failed to have an adequate clinical improvement with either drug regimen used alone or in combination, utilizing higher dosing if they failed prior combination therapies. The patients signed a consent informing them of the potential benefits and risks of DSF, including increased fatigue, brain fog/cognitive dysfunction, worsening psychiatric symptoms, liver function abnormalities, and/or increased neuropathy [96,97]. They were instructed to stop DSF immediately if there was any significant worsening in underlying symptomatology, especially neuropathic symptoms [129], and to contact our office immediately if any severe adverse effects were noted. The dose of DSF that was used ranged from a lower dose (250 mg per day or less) to a higher dose (500 mg/day) [97]. The majority of patients who took DSF in our study used doses of 250 mg/day or less to minimize the potential adverse effects and Herxheimer reactions. Disulfiram doses were slowly increased over time, increasing the dose by 62.5 mg every 1–2 weeks until reaching the target dose, which was based on efficacy and tolerance. The patients were instructed to use sodium bicarbonate or freshly squeezed lemons and/or limes to alkalize the body if Herxheimer reactions resulted from killing off *Borrelia* [130], along with N-acetyl cysteine, alpha lipoic acid, and high-dose glutathione (2000 mg). These nutraceuticals help to decrease inflammation through their effect on blocking NFKappa-B, lowering inflammatory cytokine production. Apart from the above nutritional supplements and dietary recommendations, the DSF group was also instructed to avoid ingestion and exposure to alcohol to minimize gastrointestinal complications, including nausea and/or vomiting, a known side effect of DSF toxicity [131]. All patients on tetracyclines were instructed to avoid direct sun exposure for more than several minutes to avoid the possibility of sunburn while using a 65 sun protection factor or above; to take the medication with a full 8 oz glass of water after a meal, sitting up for one hour, to avoid any reflux esophagitis; and to avoid concomitant use of dairy products and minerals within one hour of the tetracycline to avoid decreasing absorption of the medication [132].

Although both dapsone and disulfiram have demonstrated some efficacy against resistant biofilm/persister forms of *Borrelia burgdorferi* [74,94,104], up to four different biofilm agents were regularly used during the DDDCT and HDDCT protocols to improve efficacy. These included Stevia [133], Biocidin [134], essential oils including oregano, cinnamon, and clove [135], as well as peppermint oil [136]. Rarely, a fifth biofilm agent, Ethylenediaminetetraacetic acid (EDTA) suppositories (Detoxamin, 750 mg) [137], was considered the last week of HDDCT if patients had extremely severe symptoms and/or had failed prior regimens with DDDCT and HDDCT, with or without bioactive silver hydrosol (Argentyn 23), which was used for its antibacterial and synergistic effects [138]. If patients were on DSF, monolaurin and serrapeptase were substituted as biofilm agents instead of Stevia and Biocidin since they do not contain any alcohol [139,140]. Grapefruit seed extract was also used along with hydroxychloroquine for its effects against the round-body forms of *Borrelia* [141,142].

Institutional Review Board approval was not required for this work since this was a retrospective review of a convenience sample of patient charts. A convenience sample of 25 adult patient charts was chosen for inclusion in our study out of a patient population of 50 patients offered HDDCT. Protocol 1 with Table 1 and Table 2 below lists the care plan given to patients, which describes the medications and nutritional supplements that were used before, during, and after a 9-week DDDCT and HDDCT antibiotic protocol. Protocol 2 with Table 1 and Table 3 lists the two-week care plan for those who already finished an 8-week course of DDDCT at some point in the past (months, years) and had either not carried out a course of a 4-day HDDCT, a 6–7 day course of HDDCT, and/or failed to remain in remission after DDDCT and/or HDDCT.

**Protocol 1.**  *Medication and Nutritional Supplementation Protocol Sheet for Double Dose Dapsone Combination Therapy and High Dose Pulsed Dapsone Combination Therapy for CLD/PTLDS.*

Diet: Avoid aged cheese, liver, avocados, beer, wine, overripe fruit, raisins, sauerkraut, sour cream, soy sauce, yeast extracts, etc. since a low histamine diet will help protect against any increases in blood pressure while on higher doses of methylene blue (MB). Those with high blood pressure (hypertension) should have a home electronic blood pressure cuff and intermittently check their blood pressure while taking MB. Stay on a low-carbohydrate diet and avoid simple carbohydrates to avoid promoting yeast/candida overgrowth. Take tetracyclines with a full stomach and 8 oz glass of water, sitting up for one hour post-ingestion to avoid reflux esophagitis; do not mix dairy and minerals within one hour of use; and avoid more than several minutes of direct sun on tetracyclines (doxycycline, minocycline) since they can cause a sunburn. Use of a 65-sun protection factor sunscreen on all exposed areas (hands, face, etc.) is advisable, even while driving in the car.

The patient should be Glucose-6-Phosphate Dehydrogenase (G6PD) positive (an enzyme needed to help red blood cells work properly) without significant iron deficiency anemia or vitamin deficiency (B12, folate) before starting this protocol. Moreover, before instituting methylene blue, all psychiatric medications that could interact should be tapered and stopped (except benzodiazepines). The patient should provide a list of all medications and supplements for the healthcare provider to review, as medication interactions can occur (i.e., rifampin, cimetidine, clarithromycin, and methylene blue can potentially affect drug levels) and may require a dosage adjustment in medications (for example, thyroid medication may need to be increased temporarily). Coordination with a psychiatrist may be required, depending on the number and types of psychiatric medications. Table 1 below lists the nutritional supplements/support used during DDDCT and HDDCT, explaining their respective indications. Table 2, listed below, outlines the medication regimens used during DDDCT and HDDCT, delineating any weekly changes in medication and dosage, with guidelines on dosing flexibility depending on patient tolerance.

**Table 1:** Nutritional Supplements/Support for DDDCT and HDDCT.

Time Frame	Supplements/Nutritional Support	Indication
Prior to Beginning DDDCT	Biofilm agents: take cinnamon/clove/oregano oil one twice a day (Doctor Inspired Formulations, Hopkinton Drug Compounding Pharmacy, Hopkinton, MA, USA), Biocidin 2 sprays twice a day (Biocidin Botanicals, Aptos, CA, USA), Stevia 15 drops twice a day (NutraMedix, Jupiter, FL, USA) and peppermint oil capsules one twice a day (Infuserve America Compounding Pharmacy, St. Petersburg, FL, USA). If you have had severe/resistant symptoms, speak to your provider about adding on Argentyn 23, 1 teaspoon twice a day (Natural Immunogenics, Sarasota, FL, USA) during the last month of the protocol with or without EDTA suppositories (Detoxamine, Draper, UT, USA) the last week of the protocol for extra biofilm support [137,143].	Biofilm support
	Probiotics: Orthobiotic (Ortho Molecular Products, Woodstock, IL, USA), *Saccharomyces boulardii* (Ortho Molecular Products, Woodstock, IL, USA), Theralac (Master Supplements, Victoria, MN, USA) are all taken twice a day first thing in the morning and last thing before bed, along with ½ a packet of Probiomax 350 billion (Xymogen, Orlando, FL, USA) once a day (this can be used twice a day if there are any loose stools).	Microbiome support
	Detoxification/Inflammatory Support: N-Acetyl Cysteine (NAC) 600 mg twice a day (Xymogen, Orlando, FL, USA), glutathione 4 capsules (250 mg each) twice a day (Ortho Molecular Products; or Essential Pro Glutathione, Wellness Pharmacy, Birmingham, AL, USA), Alamax (alpha lipoic acid, Xymogen) 600 mg, one twice a day, curcuplex (Xymogen) 500 mg, one twice a day, sulforaphane glucosinolate (Oncoplex ES, Xymogen) 100 mg, one twice a day, vitamin C 1–2 g twice a day (Xymogen), vitamin E 300 IUs twice a day (Designs for Health, Suffield, CT, USA), NADH (ENADA Nutraceuticals, Las Vegas, NV, USA) 5 mg twice a day; please keep Alka-Seltzer gold or sodium HCO3 (bicarbonate) at home which can be used as needed for temporary increases in underlying Lyme disease symptoms, i.e., severe Herxheimer reactions [130], which is used with 2 g of glutathione all at once, up to three times daily until the Herxheimer reaction resolves [50,144]. You can also use up to 2000 mg of glutathione 3× per day if there are symptoms of methemoglobinemia [118] (blue hands, blue lips, headaches, fatigue, shortness of breath) although the symptoms would be unusual using higher dose methylene blue at a dose of 300 mg twice a day, as it helps keep down significantly elevated methemoglobin levels.	Detoxification/ Inflammatory Support
Weeks 1–4 of DDDCT (slowly increasing dapsone from 25 mg per day to 100 mg per day)	Continue biofilm, microbiome and detoxification/inflammatory support listed above. Add Folic acid support once starting dapsone: two tablets of L-methyl folate 15 mg each, twice a day month one (weeks 1–4), for a total of 60 mg of L-methyl folate per day (Xymogen, Folafy-ER). Folic acid supplementation can be taken at the same time as all antibiotics, twice a day, after breakfast and dinner, with a full stomach. Probiotics should be taken away from antibiotics, i.e., first thing upon awakening, and last thing at night before bedtime. Add B12 support (Methyl protect, one a day, Xymogen) and over the counter (OTC) iron (Fe), between 45–325 mg per day (at lunch, away from antibiotics). Any mineral supplements (magnesium, calcium, zinc, iron, copper, etc.) should be taken at least one hour away from antibiotics to avoid interfering with absorption of tetracyclines.	Addition of Folic acid, B12 and iron helps reduce dapsone induced anemia
Week 5–8 DDDCT (100 mg BID)	Continue biofilm, microbiome and detoxification/inflammatory support listed above. Continue B12 and iron support, as listed above. Folic acid dosing doubles on DDDCT: use 4 tablets of L-methyl folate 15 mg each, twice a day, for a total dose of 60 mg of L-methyl folate twice a day (120 mg total). Keep Alka-Seltzer gold or sodium HCO3 (bicarbonate) at home which can be used as needed for temporary increases in underlying Lyme disease symptoms, i.e., severe Herxheimer reactions [130], which is used with 2 g of glutathione all at once, up to three times daily until the Herxheimer reaction resolves [50,144]. You can also use up to 2000 mg of glutathione 3× per day if there are symptoms of methemoglobinemia [118] (blue hands, blue lips, headaches, fatigue, shortness of breath) although the symptoms would be unusual using higher dose methylene blue.	Antioxidant support to help lower methemoglobin levels, along with biofilm, microbiome, and detoxification/inflammatory support. Extra folic acid helps reduce dapsone induced anemia
Week 9 (HDDCT)	Continue biofilm, microbiome and detoxification/inflammatory support listed above. Continue folic acid, B12 and iron support, as listed above. Depending on laboratory results week 9, adjust glutathione doses and/or folic acid dosing. This will be based on levels of anemia, and methemoglobin levels. L-methyl folate doses can be increased to 5–6 tablets twice a day week 9 if needed for any increases in anemia from higher dose dapsone. Glutathione dosing can increase to 2000 mg TID, if there are any increases in methemoglobin despite methylene blue dosing at 300 mg PO BID.	Biofilm, microbiome, inflammatory, detoxification, hematological support
Weeks 10–14 (first month off dapsone)	Continue biofilm, microbiome and detoxification/inflammatory support listed above. Continue folic acid, B12 and iron support, as above. Folic acid doses for L-methyl folate may be slowly decreased from 4–5 twice a day (by week 11) to 3 twice a day (week 12) to 2 twice a day (weeks 13–14, i.e., 60 mg of L-methyl folate per day) depending on the CBC and rapidity of reversal of anemia off dapsone. Individualized dosing for folic acid will depend on the level of anemia; a follow-up CBC and CMP with haptoglobin levels should be done 5–7 days post dapsone combination therapy, especially if any sudden unexpected drops in hemoglobin levels were noted the week on HDDCT (week 9), and/or if any other significant hematological abnormalities were noted. If a CBC and CMP are stable one week post dapsone, repeat laboratory testing week 2–3 weeks later to adjust folic acid dosing. Add a one month mitochondrial regeneration program weeks 10–14: This includes: ATP 360, 3 capsules once a day (Researched Nutritionals), ENADA (NADH) one twice a day; carnitex (Xymogen) two twice a day (not for those with alpha gal allergy), CoQ Power 400 mg twice a day (Researched Nutritionals), Cardio Ribose (Researched Nutritionals), one scoop twice a day, along with Mitoprime (Xymogen) one a day, and Mito NR (Designs for Health, Suffield, CT, USA) two a day. Mitochondrial support has been shown to be helpful in certain patients with a chronic, fatiguing illness. You can stop the mitochondrial supplements after one month post high dose dapsone combination therapy.	Continue to replace healthy GI bacteria with microbiome support 1 month post DDDCT and HDDCT. Continue antioxidant and anti-inflammatory support post DDDCT and HDDCT Mitochondrial support can reverse potential mitochondrial damage from high levels of free radicals/oxidative stress during therapy
Weeks 15–19 (2nd month post DDDCT and HDDCT	Lower biofilm support to once a day; Lower microbiome support to once a day. B12 and iron supplementation may be stopped if no deficiencies noted. Antioxidant/anti-inflammatory support with NAC, alpha lipoic acid, glutathione, Curcuplex and sulforaphane glucosinolate (Oncoplex ES) may be continued if ongoing sources of inflammation are present, including but not limited to ongoing infections, environmental toxins (mold, heavy metals), or if needing COVID support. Folic acid dosing is based on the rapidity of reversal of the CBC off dapsone. Patients may require staying on a lower dose of L-methyl folate (15 mg BID) depending on the CBC.	

Abbreviations: Complete blood count (CBC), Comprehensive metabolic profile (CMP), Double dose dapsone combination therapy (DDDCT), Ethylenediaminetetraacetic acid (EDTA), High dose dapsone combination therapy (HDDCT), N-acetyl cysteine (NAC), nicotinamide adenine dinucleotide with hydrogen (NADH),

### Medication Regimen for DDDCT and HDDCT

Table 2, listed below, outlines the medications used during DDDCT and HDDCT. Methylene blue (MB) will be prescribed orally to help decrease one of the major side effects of higher-dose dapsone therapy, methemoglobinemia (decreased oxygen carrying capacity) [106]. Side effects of MB include blue/green urine, occasional urinary discomfort, potential increases in blood pressure, serotonin toxicity (if taking an SSRI or certain psychiatric medications), and interference with the light emission of a pulse oximeter, leading to incorrectly low oxygen saturation measurements [145]. For those needing phenazopyridine (Pyridium) to decrease urinary tract discomfort, a potential side effect of higher doses of methylene blue, it can be prescribed at a dose of 200 mg three times a day as needed during the last weeks of the protocol. The patient should inform their healthcare provider if they have any urinary discomfort requiring this medication. Moreover, patients may require Zofran (ondansetron) for nausea [146] while on high-dose dapsone. Nausea (and rarely vomiting) may occur when the dose of dapsone is raised to 200 mg twice a day. The use of Zofran requires having a normal EKG and QT interval, as ondansetron may cause EKG interval changes such as QTc elongation, which can potentially cause cardiac arrhythmias [146]. A baseline copy of an EKG is therefore required, and if normal, we suggest, while on Plaquenil and Zithromax, medications that can also potentially cause QTc prolongation [114], taking a dose of Zofran and then going for a repeat EKG to ensure that the QT interval remains within normal limits. An EKG requisition should therefore be provided to the patient before and during DDDCT and HDDCT.

**Table 2:** Medication Regimen for DDDCT and HDDCT.

Time Frame	Medication	Nutritional Support	Laboratory/EKG
Prior to beginning DDDCT	Add medications gradually to ensure GI tolerance. For example, start with Plaquenil (hydroxychloroquine) 200 mg twice a day after meals, Nystatin 500,000 U tablets twice a day, and minocycline (or doxycycline) 100 mg twice a day. Lower-dose minocycline or doxycycline (50 mg twice a day) can be initiated if there is a history of GI intolerance to tetracyclines. If there is no issue with GI tolerance in an adult patient, add rifampin (300 mg BID). Rifabutin 150 mg PO BID may be substituted for rifampin if you are intolerant to rifampin. After several days, then add pyrazinamide (which is dosed by body weight): up to 55 kg = 1000 mg once a day (2 pills of 500 mg once a day); 56–75 kg = 1500 mg once a day (3 pills once a day); over 76–90 kg = 2000 mg once a day (4 pills once a day). The 8-week DDDCT protocol starts when all antibiotics are on board and tolerated (which may add an extra week to the protocol). Rifampin may affect medication levels. Perform a drug interaction check. Adjust doses	See Table 1 regarding doses of biofilm agents, probiotics, detoxification, and inflammatory support.	Patients should be G6PD positive without B12, folate, or iron deficiency before starting dapsone EKG should be normal
Week 1	Plaquenil 200 mg PO BID, doxy (or minocycline) 100 mg PO BID, rifampin 300 mg PO BID, Nystatin 500,000 U tablets, 2 PO BID. Start dapsone 25 mg one PO QD, taken at the same time as the other antibiotics. Add folic acid support: Leucovorin (folinic acid) 25 mg two twice a day (50 mg twice a day) along with 15 mg of L-methyl folate, 2 twice a day. See Table 1 for details.	Add Folic acid support once starting dapsone.	
Week 2	Same doses of the above medication, but increase dapsone to 25 mg PO BID (50 mg/day). Clotrimazole (Mycelex) troches (10 mg QID prn) may be used at any time during the protocol if, despite a strict sugar-free/yeast-free diet, any signs of yeast/Candida arise on the tongue.	See Table 1	
Week 3	Same doses of the above medication; increase dapsone to 50 mg in the am, 25 mg in the pm for a total of 75 mg per day.	See Table 1	CBC, CMP is ordered at the end of week 3 with a baseline MetHb
Week 4	Same doses of the above medication; increase dapsone to 50 mg in the morning and 50 mg in the afternoon for a total of 100 mg of dapsone per day. Order 100 mg of dapsone tablets from the pharmacy for week 5 (90 tablets) and renew all of the above medications. Start methylene blue 50 mg twice a day week 4. This is taken with high-dose antioxidants to help reverse methemoglobin levels. See Table 1. This would include 1000 to 2000 mg of glutathione twice a day, vitamin C 1–2 g twice a day, vitamin E 300 IU twice a day, and NADH 5 mg twice a day. Consider starting cimetidine 400 mg twice a day if there has been a history of any significantly elevated methemoglobin levels proceeding into month two. If cimetidine is required due to elevated methemoglobin levels, lower Plaquenil (hydroxychloroquine) once a day and check drug interactions for potential medication interactions. If any significant Herxheimer reactions occur at 100 mg of dapsone that do not resolve with alkalizing and high dose glutathione, or if there is any evidence of unexpected anemia (greater than a 2–3 g drop in hemoglobin) with unexpected rises in methemoglobin (greater than 5%) proceeding into week 4, the dose of dapsone can be temporarily held for a week, increasing Leucovorin to 4 tablets twice a day (100 mg PO BID) with 4, 15 mg L-methyl folate twice a day (60 mg PO BID) until the anemia improves and/or the methemoglobin level decreases. Then restart dapsone at 100 mg per day.	See Table 1 for nutritional support.	
Week 5	Take dapsone 100 mg, 1 dose twice a day (this is officially the start of one month of double-dose dapsone). Add 250 mg of azithromycin (Zithromax) twice a day with a full stomach. Clarithromycin (Biaxin) can alternatively be used as a macrolide instead of Zithromax if insurance coverage requires a different medication. Increase Leucovorin to 25 mg, 4 tablets PO BID (100 mg BID) with 15 mg of L-methyl folate, and 4 tablets PO BID (60 mg BID) for a total dose of 320 mg of folic acid. Increase methylene blue (MB) to 100 mg PO BID × 3 days, then increase MB to 150 mg PO BID. The total dose of MB at the end of week 5 is 300 mg per day. All other medications and nutritional supplements remain the same. Check an EKG and rule out QT prolongation on hydroxychloroquine and azithromycin with one dose of 8 mg of ondansetron (Zofran). If the EKG is stable, you may use ondansetron Q 8 h prn for nausea. Use phenazopyridine (Pyridium) 200 mg PO TID prn for any urinary burning/discomfort on MB.	See Table 1 for nutritional support	CBC, CMP, MetHb, haptoglobin end of week 5 & q week. Check hormones (thyroids, etc.) on rifampin and adjust prn. EKG
Week 6	Increase methylene blue to 200 mg PO BID. All other medications remain the same. May increase methylene blue to 250 mg PO BID if methemoglobin level is >5% despite using antioxidants in Table 1. May add cimetidine if needed for elevated MetHb	See Table 1	CBC, CMP, MetHb, haptoglobin
Week 7	Increase methylene blue to 250 mg PO BID. All other medications remain the same. May increase methylene blue to 300 mg PO BID if methemoglobin level is >5%. If CBC, CMP, MetHb is stable, continue protocol. May hold dapsone temporarily if any unexpected drops in hemoglobin from baseline (average drop in Hb is 3.5–4 g on DDDCT, which is to be expected).	See Table 1	CBC, CMP, MetHb, haptoglobin Follow BP QD on MB
Week 8	Increase methylene blue to 300 mg PO BID. This is the final dose adjustment for MB. All other medications remain the same. Dapsone may cause low-grade hemolysis, but if CBC, CMP, and MetHb are stable, continue the protocol. In rare cases, higher doses of MB may result in increased hemolysis. Hold dapsone, increase folic acid dosing, and lower MB to 200 mg PO BID if any unexpected drops in Hb result.	See Table 1	CBC, CMP, MetHb, haptoglobin
Week 9 (HDDCT)	Increase dapsone to 200 mg PO BID for 4 days in a row if treating CLD without evidence of co-infections such as *Bartonella*, or increase dapsone to 200 mg PO BID × 6 days in a row if treating *Bartonella*. This is a high-dose dapsone combination therapy. All other medications remain the same. Use ondansetron (Zofran) 8 mg q 8 h prn for any nausea/vomiting. Check labs after 3 and 5 days on HDDCT and adjust dosing based on symptoms and laboratory results. Stop dapsone if the hemoglobin level is >20% and if there is an unexpected drop in hemoglobin > 1–2 g from the prior CBC, hold dapsone, increase folic acid dosing, and lower the MB to 200 mg PO BID.	See Table 1	CBC, CMP, MetHb, and haptoglobin on days 3 and 5 of HDDCT Follow BP QD on MB
Week 10, First week off HDDCT	Stop all antibiotics including tetracyclines (doxycycline or minocycline), rifampin (or rifabutin), pyrazinamide, azithromycin (or clarithromycin), and hydroxychloroquine (Plaquenil). Remain on same doses of Nystatin, Leucovorin, L-methyl folate, all probiotics, biofilm support, and nutritional support. Taper MB. First day off dapsone take 300 mg PO BID, then decrease MB to 200 mg BID for 2 days, 100 mg BID for 2 days, 50 mg PO BID for 2 days, and then stop methylene blue.	See Table 1 Add mitochondrial support	CBC, CMP, MetHb, haptoglobin 4 days post-HDDCT
Week 11	Increase folic acid dosing prn if any unexpected decreases in hemoglobin occur. For example, may increase Leucovorin and L-methyl folate by 1–2 tabs BID for 1 week	See Table 1	Repeat CBC if unexpected decrease in Hb
Week 12	Decrease folic acid dosing if CBC is stable	See Table 1	
Week 13	Decrease folic acid dosing if CBC is stable	See Table 1	CBC, CMP
Weeks 14–18, 2nd mo.	Continue to decrease folic acid and nutritional support. If the patient is in remission, no further pulses of HDDCT are required. If *Bartonella* is still active, consider a 2 week HDDCT pulse (14 days), i.e., Protocol 2, q 6–8 weeks once labs return to normal	See Table 1	CBC, CMP end of month 2

**Protocol 2.**  *Two-week HDDCT Protocol Medication and Nutritional Supplementation Sheet for CLD/PTLDS.*

This protocol is primarily for patients who have completed an 8-week DDDCT in the past (months or years) with or without a course of HDDCT and have ongoing resistant symptoms attributable to tick-borne disease (i.e., *Bartonella*). 

For those who have already completed a double-dose dapsone protocol and are now looking to perform a high-dose dapsone pulse protocol for *Bartonella* and are not on any antibiotics, it is advisable to slowly increase and add antibiotics every few days during the first week to ensure GI tolerance. See Table 3 below for instructions on how to implement the two-week HDDCT protocol.

**Table 3:** Two Week HDDCT Medication Regimen.

Time Frame	Medication	Nutritional Support	Laboratory/EKG
Week 1	Beginning on a Monday, start with Plaquenil (hydroxychloroquine) 200 mg twice a day, minocycline 100 mg twice a day (or doxycycline 100 mg PO BID), Nystatin 500,000 units, two twice a day, and after two days add rifampin (Wednesday), 300 mg twice a day; then after one day (Thursday), add pyrazinamide (PZA) 500 mg tabs, 2–4 once a day (the dose is dependent on body weight (see Table 2); then after one more day (Friday), add azithromycin (Zithromax) 250 mg twice a day. All antibiotics are taken on a full stomach after breakfast and dinner. The methylene blue dosage for week one of this protocol is 50 mg twice a day × 2 days (Monday, Tuesday), then 100 mg twice a day for 2 days (Wednesday, Thursday), then 200 mg twice a day for 3 days (400 mg by days 6–7). At the end of week one, the patient will therefore be on Plaquenil, minocycline (or doxycycline), rifampin (or rifabutin), PZA, Zithromax, Nystatin, and methylene blue. See Table 1 for nutritional support with biofilm agents, probiotics, detoxification, and inflammatory support. Follow a low-histamine diet as per Protocol 1. All psychiatric medications that can interact with methylene blue (MB) must be stopped at least one week before using MB and several weeks afterwards to avoid potential side effects.	See Table 1	CBC, CMP, and Methemoglobin levels should be normal before starting HDDCT EKG should be WNL; check QT intervals on Plaquenil, azithromycin + ondansetron if not previously conducted
Week 2	Dapsone starts week two, and day 8, is added at a dose of 100 mg twice a day for 1 day (double dose dapsone). This will be taken with Leucovorin 25 mg, 4, twice a day, with L-methyl folate 15 mg (Folafy-ER) 4 twice a day along with all antibiotics and nutritional supplements from week 1. Rifampin will be increased to double dose (300 mg, 2 twice a day) at the beginning of week 2 if liver functions are WNL. For a 4 day HDDCT pulse, days 9–12 you will take high dose dapsone × 4 days (200 mg twice a day). This is primarily for those with CLD/PTLDS without associated co-infections (i.e., *Bartonella*). For a 6-day HDDCT pulse, i.e., for those with active intracellular co-infections (e.g., *Bartonella*), take high-dose dapsone (200 mg twice a day) for 6 days, days 9–14, with the same doses of folic acid. The last week, days 8–14, the methylene blue dose is increased to 300 mg twice a day once starting dapsone and continued at that dosage for as long as the patient remains on HDDCT. For those needing phenazopyridine (Pyridium) to decrease urinary tract discomfort, it can be prescribed at a dose of 200 mg three times a day while on methylene blue. Ondansetron 4–8 mg Q8 prn may be used prn for nausea and/or vomiting if QT intervals are WNL	See Table 1	CBC, CMP, Methemoglobin, and haptoglobin levels are drawn on day 10 and day 12 to rule out any significant hematological and/or laboratory changes
Week 3	Stop all antibiotics including tetracyclines (doxycycline or minocycline), rifampin (or rifabutin), pyrazinamide, azithromycin (or clarithromycin), and hydroxychloroquine. Remain on same doses of Nystatin, Leucovorin, L-methyl folate, all probiotics, biofilm support and nutritional support. Increase folic acid dosing prn if any unexpected decreases in hemoglobin occur post high dose dapsone, weeks 3–4. For example, may increase Leucovorin and L-methyl folate by 1–2 tabs BID for 1 week, then begin a slow taper off folic acid based on the results of the CBC. Taper MB. First day off dapsone take 300 mg PO BID, then decrease MB to 200 mg BID for 2 days, 100 mg BID for 2 days, 50 mg PO BID for 2 days, and then stop methylene blue. Start mitochondrial support.	See Table 1	Repeat CBC, CMP, Methb and haptoglobin levels on days 17–18, 3–4 days post dapsone
Week 4	Continue with a mitochondrial regeneration protocol post-treatment for a total of 4 weeks (weeks 3–7). ATP 360, 3 a day (Researched Nutritionals), ENADA, one a day; carnitex, 2 twice a day (Xymogen, not for those with alpha-gal allergy); CoQ Power, 2 times a day (Researched Nutritionals); Cardio Ribose, one scoop twice a day; along with Mitoprime (Xymogen), 1 a day; and Mito NR (Designs for Health), 2 a day. Mitochondrial support has been shown to be helpful in some patients with chronic, fatiguing illnesses. Stop the mitochondrial supplements after one month of high-dose dapsone combination therapy. Continue to slowly taper folic acid dosing if the CBC is stable.	See Table 1	
Weeks 5–6	Finish the mitochondrial regeneration protocol. Continue to taper folic acid. Continued folic acid support will depend on the CBC 3 weeks post-HDDCT. Taper biofilm agents, probiotics, detoxification/inflammatory support as per Table 1 post-dapsone.	See Table 1	Repeat CBC, CMP 3 weeks post HDDCT

## 3. Results

Among the 25 participants, 15 were men and 10 were women. The age range was between 18 and 63 years old (Mean = 42.04, SD = 14.2). A total of 68% were less than 50 years old, and 32% were older than 50 years old. The mean length of illness was 14.7 years. A total of 24% of patients (*n* = 6) had been ill for more than 20 years, 40% (*n* = 10) had been ill between 10 and 20 years, 24% (*N* = 6) had been ill between 5 and 9 years, and 12% (*n* =3) had been ill for less than 5 years in duration. Eight percent of patients (*N* = 2) had physician-diagnosed EM rashes and therefore met the criteria for PTLDS. Another patient had multiple red rashes 22 years ago, prior to getting ill; however, the diagnosis of Lyme disease had not been established by a physician. Among these 25 participants with CLD/PTLDS, 44% (11/25) had CDC-positive IgM Lyme immunoblots, and one patient out of 25 (4%) had at least one co-infection; 2 patients (8%) had at least 2 co-infections; and 22 out of 25 patients (88%) had three or more co-infections. Five patients (20%) had positive titers for *Relapsing Fever Borrelia* (TBRF), 3 patients (12%) had antibody titers to *Borrelia miyamotoi*, and 16/25 patients (64%) had exposure to *Babesia* species, with several patients having more than one species present (9 patients with *Babesia microti*; 10 *Babesia duncani*; 2 *Babesia odocoilei* (FISH); 1 *Babesia divergens*). Five out of 25 patients (20%) had evidence of active *Babesia*, as they were *Babesia* FISH positive. Twenty-one patients (84%) tested positive for one or more *Bartonella* species. These included: 11 patients with *Bartonella henselae*; 7 with *Bartonella quintana*; 6 with *Bartonella elizabethae*; 6 with *Bartonella vinsonii* subspecies; 1 with *Bartonella koehlerae*; 1 with *Bartonella bacciliformis*; and 4 with *Bartonella* spp. (genus) without specific spp. identification. Seventeen patients (68%) had evidence of active *Bartonella*, with 11 patients (44%) being positive by *Bartonella* FISH and 7/25 (28%) having evidence of active *Bartonella* via elevated vascular endothelial growth factor (VEGF) or changes in striae. Other co-infections included 20% of patients with evidence of *Ehrlichia* (5/25), 16% (4/25) with *Anaplasma*, 24% (6/25) with low positive tularemia titers, 16% (4/25) with *Rickettsia* spp., 16% (4/25) with low positive titers to *Brucella* spp., 72% (18/25) with prior exposure to *Mycoplasma pneumonia*, 12% (3/25) with *Chlamydia pneumonia*, 96% (24/25) with evidence of exposure to herpes viruses 1, 2, 6, and EBV (HSV-1, 2, HHV-6, EBV); 20% (5/25) with Cytomegalovirus (CMV); 8% (2/25) with West Nile virus (WNV) exposure; 52% (13/25) with COVID exposure before, during, or after DDDCT and HDDCT; 32% (8/25) were positive to Coxsackie/Parvovirus B-19; and 60% (15/25) had evidence of *Candida*/hypoglycemia.

**Figure 1:**
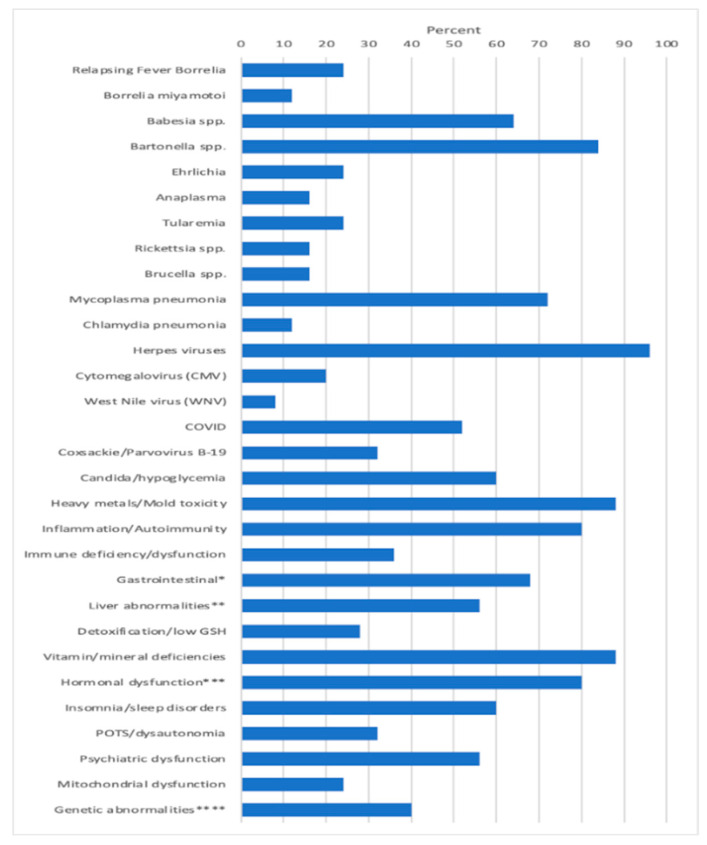
MSIDS Variables Present Among 25 Patients with CLD/PTLDS on DDDCT/HDDCT.

Apart from these infections, other MSIDS variables present which potentially affected underlying symptomology included patients having evidence of 6 different mold toxins (Ochratoxins, 7/25 patients (28%); Trichothecenes, 11/25 (44%); Aflatoxins, 8/25 (32%); Gliotoxins, 11/25 (44%); Zearalenone, 9/25 (36%); Citrinin/Dihydrocitinone, 1/25 (4%); exposure to 8 heavy metals (Mercury (Hg) 10/25 patients (40%); Lead (Pb), 11/25 (44%); Arsenic (As), 3/25 (12%); Tin (Sn), 1/25 (4%); other: Cesium (Ce), Uranium (Ur), Thallium (Thal), Antimony (Sb), 7/25 (28%); Inflammation/Autoimmune markers: 20/25 (80%); Immune Deficiency (CVID), 5/25 (20%); Other immune abnormalities (i.e., CIDP, ↓ CD4 counts, ↓ Total Natural Killer (NK) cells, ↑ IgM antibodies), 5/25 (20%); Leaky Gut/Food Sensitivity/Mast Cell Activation Syndrome (MCAS), 15/25 (60%): GI Microbiome Abnormalities, Gastroparesis, 6/25 (24%); Liver Abnormalities, i.e., Non-alcoholic Steatohepatitis (NASH), ↑ LFT’s, 11/25 (44%); Liver Abnormalities, Low Alkaline Phosphatase, 6/25 (24%); Detoxification Abnormalities, Low glutathione (GSH), 7/25 (28%); Vitamin and mineral deficiencies, 22/25 (88%); Hormonal abnormalities, low adrenal function, 20/25 (80%); Hormonal abnormalities, low testosterone (men), 6/25 (24%); Hormonal abnormalities, absent pituitary function, 1/25 (4%); Hormonal abnormalities, elevated insulin/Polycystic Ovarian Syndrome (PCOS), 1/25 (4%), Hormonal abnormalities, low thyroid function, 7/25 (28%); insomnia/sleep disorders (2 with Obstructive Sleep Apnea, i.e., OSA), 17/25 (68%); POTS/dysautonomia, 8/25 (32%); Psychiatric disorders (Depression, Anxiety, Obsessive Compulsive Disorder (OCD), PTSD, Schizophrenia), 14/25 (56%); Mitochondrial dysfunction, 6/25 (24%); and genetic abnormalities (MTHFR, HLA DR4), 10/25 patients (40%). Figure 1 illustrates the percentage of patients among the 25 participants with overlapping MSIDS variables potentially affecting the clinical outcome.

Treatment results: A total of 25 out of 25 patients (100%) had improvement in their tick-borne symptoms. Of 23 patients who completed a full 8-week course of DDDCT prior to one or several courses of HDDCT, 7/23 (30.5%) had a full resolution of their Lyme and tick-borne symptoms for 3 months or longer, even if there was prior evidence of co-infections, including *Babesia* and/or *Bartonella*. Three out of 23 patients (13%) also had a full resolution of their Lyme and tick-borne symptoms for 1½ months or longer, having recently completed the full protocol 6 weeks prior; among those three patients, patient number 10 (see Table 4 below for details of all 25 patients) was *Bartonella* FISH positive and remained in remission for 2 months after one course of DDDCT and three-four-day pulses of HDDCT; however, then relapsed during her menstrual cycle for several days, becoming 100% asymptomatic again after doing a 6-day HDDCT pulse.

A total of 2/25 patients (8%) were EM rash positive with a history of PTLDS, and 1/2 (50%) went into remission for 3 months or longer. Patient 5 had an EM rash 21 years prior, and although DDDCT in 2019 helped to relieve the vast majority of symptoms for several years, it was not until completing a 4-day course of HDDCT 3 years later, in December 2022, that residual fatigue, joint pain, irritability, insomnia, and word recall issues resolved 100% for 6 months or longer.

Of the sixty-four percent (16/25) of patients with babesiosis, 18.8% (3/16) were in full remission. None of the 6 patients who were *Babesia* FISH positive remained in remission, although 4/6 of them (66.6%) were greater than 30% improved and 2 patients were somewhat improved (range of 0–20%). The last patient, patient number 25 (see Table 2), was functioning however at 90% of normal, and patient number 18, who was over 18 months in full remission with all physical symptoms, including symptoms of Babesiosis, had a cognitive relapse at the end of 2022, which may or may not have been due to tick-borne disease.

Six out of 25 patients (24%) took DSF in doses varying between 62.5 mg alternating with 125 mg, up to 500 mg per day, for up to 6 months at a time, during their course of therapy with dapsone for the treatment of Lyme disease and *Babesia*. It was well tolerated in 5/6 patients (83.3%), and DSF needed to be stopped in one patient due to a neuropsychiatric flare with anxiety. Disulfiram was taken with dapsone for the treatment of resistant *Babesia* in patient number 4 (*B. duncani*), patient 13 (*B. microti*, *B. duncani*, *Babesia* FISH+), patient 17 (*Babesia duncani*, *B. odocoilei* FISH+), patient 18 (*Babesia* FISH+), patient 21 (*Babesia duncani*), and patient 25 (*B. duncani*), and the combination therapy was helpful in relieving symptoms of babesiosis, i.e., symptoms of sweats, chills, flushing, air hunger, and/or a cough. Regimens for *Babesia* apart from using dapsone and DSF included rotations of atovaquone with or without azithromycin and/or clindamycin [147,148], atovaquone/proguanil (Malarone) [149], tafenoquine [150], with or without ivermectin [151] and Malarone, artemether/lumefantrine (Coartem) [152], low dose mefloquine [153], and botanical protocols, i.e., artemisinin (*Artemisia annua*) [154], with or without cryptolepis (*Cryptolepis sanguinolenta*) [155], as well as Chinese skullcap (*Scutellaria baicalensis*) and Japanese knotweed (*Polygonum cuspidatum*) [156]. Five out of six patients (83.3%) who took dapsone with DSF with rotations of these antimalarial medications and botanical protocols during their course of therapy had a resolution of their *Babesia* symptoms, except for patient 21. None of the patients who had active *Babesia* (FISH+) who took dapsone and DSF with or without the above antimalarial medications and botanicals, however, remained in full remission.

Among 21/25 patients (84%) who had a history of *Bartonella* exposure, 100% improved their underlying symptomatology after at least one course of DDDCT and one or several courses of HDDCT pulses (4 days or 5–7 days). Four out of 21 patients (19%) went into long-term remission for 3 months or longer; 3/21 patients (14.3%) went into full remission for up to 6 weeks; and 14/21 patients (66.6%) improved. Of those patients not in full remission, symptom improvement for all *Bartonella* patients was at levels ranging from 15% to greater than 70%. Examining the differences in treatment response between a 4 day pulse and a 5–7 day HDDCT pulse for the treatment of *Bartonella*, 6/21 patients (28.5%) who took at least one course of DDDCT and one 5–7 day HDDCT pulse went into remission, and no patients who took DDDCT with one or several 4 day HDDCT pulses went into remission if *Bartonella* was active. An 8-week DDDCT followed by a 5–7-day HDDCT pulse was therefore superior to a shorter 4-day HDDCT pulse for those with CLD who also had chronic active *Bartonella*. Among the 8 patients with CLD and *Bartonella* who took one or several 4-day HDDCT pulses without a 5–7 day HDDCT pulse, there was nevertheless significant improvement in underlying symptomatology. Symptoms of bartonellosis that improved with either DDDCT followed by 4 day or 5–7 days HDDCT pulses with MB included 8 major Lyme symptoms, i.e., fatigue, joint pain, myalgias, neuropathy, headaches, sweats, chills, cognitive functioning, mood, and insomnia, but also included improvement in pain in the soles of the feet, striae, and *Bartonella* pseudo seizures (which may have been complex partial seizures) [67].

An 8-week DDDCT followed by a 4-day HDDCT pulse was, however, effective in putting several patients into long-term remission with CLD/PTLDS if there was no evidence of exposure to *Bartonella*. Three patients who did one course of DDDCT and one course of HDDCT for 4 days (patients 5, 6, and 7) went into long-term remission, and none of them had evidence of exposure to *Bartonella*. The 4-day HDDCT pulse after 8 weeks of DDDCT was therefore adequate for treating chronic LD/PTLDS if *Bartonella* was not present. All patients except patient 23 took PZA during DDDCT + HDDCT.

**Figure 2:**
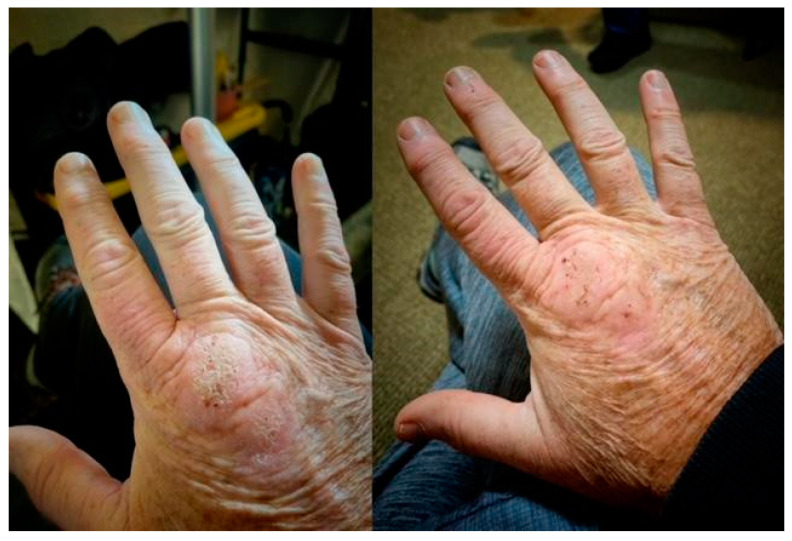
*Bartonella* Granulomas Pre and Post HDDCT.

Methylene blue (MB) dosage, apart from the length of HDDCT pulses, affected the efficacy and tolerability of the protocol. Earlier regimens of 4-day HDDCT used 100 mg of MB PO BID (twice a day). These doses were subsequently increased after patients relapsed post-therapy, with evidence of increased methemoglobin. The MB dose was therefore increased to 150–200 mg PO BID, and if symptom relapses persisted with elevations in methemoglobin, the dose was increased to a final dose of 300 mg PO BID while using HDDCT. Examples of increased efficacy of higher doses of MB were seen in patients 10 and 24, who were both *Bartonella* FISH positive, and patients 14 and 21, who had elevated VEGFs, consistent with active *Bartonella*. Patient number 10 did her first HDDCT pulse with 100 mg of methylene blue (MB) PO BID, and she was one month symptom-free before relapsing. She therefore did a second 4-day HDDCT pulse, using 200 mg PO BID of MB, which improved symptomatology but was insufficient to put her in full remission. A third HDDCT pulse for 4 days with 300 mg PO BID of MB was therefore instituted, which resulted in her being symptom-free for 2 months, her longest time without a relapse. She recently finished her first 6-day HDDCT pulse using 300 mg PO BID of MB after doing 3/4-day HDDCT pulses with lower doses of MB and has remained 100% symptom-free (approximately 2 more months in remission). Patient number 24 suffered from Lyme disease (C6 ELISA positive), active *Babesia* (FISH+), active *Bartonella* (FISH+), and multiple MSIDS abnormalities, including hypopituitarism, POTS, CVID (on IVIG), small fiber neuropathy, severe resistant insomnia, anxiety with PTSD, inflammation, multiple vitamin and mineral deficiencies, and Morgellons’s disease. She had conducted 2 courses of DDDCT, 3 courses of HDDCT for 4 days, and one course of HDDCT for 6 days. Earlier courses of pulse HDDCT were for 4 days and used a lower dose of MB (100–150 mg PO BID). The last 6-day HDDCT pulse used 300 mg of MB. Although there was temporary improvement in symptomatology with DDDCT and three four-day pulses of HDDCT with lower dose MB, it was not until the last course of six days of HDDCT with 300 mg PO BID of MB that she noticed persistent and sustained improvement in energy, myalgias, neuropathy, and cognitive impairment for five months. Patient 21, after 3 courses of DDDCT and 3 courses of HDDDT for 4 days, where the last round used a higher dose of MB at 300 mg BID, resulted in sustained improvements in energy/endurance, headaches, decreased joint pain, decreased neuropathy, dizziness, and some movement of paralyzed muscles of the lower extremities during the past 4 months. The lower doses of MB used in 4-day HDDCT pulses did not result in sustained improvement or movement of paralyzed muscles in the lower extremities. Finally, patient 14 had 3 spp. of *Bartonella* (Bh, Be, and Bv), an elevated VEGF, implying active *Bartonella*, and a *Bartonella* granuloma on her right hand. She also suffered from severe depression and anxiety with POTS, similar to the other patients with multiple species of *Bartonella* and *Babesia*. After 2 courses of DDDCT and one 6–7 day course of HDDCT with minocycline, rifampin, azithromycin, and pyrazinamide in December 2022, along with a higher dose of MB at 300 mg PO BID, she noticed a marked improvement in clinical symptomatology, going from 25% to 87% of normal functioning, with improvements in fatigue, chills, joint pains, and cognition. Her joint pain and swelling decreased, and the *Bartonella* granuloma on her right hand decreased in size by 80%. See Figure 2.

Age, length of illness (LOI), and gender were also evaluated to determine their effect on the treatment outcome. Neither age, LOI, nor gender affected remission rates or levels of improvement. Among 17 patients less than 50 years of age, 4 patients went into full remission for 3 months or longer; one patient went into remission for 6 weeks or longer; 5 patients improved greater than 50% of normal; 1 patient improved by greater than 45% of normal; 2 patients improved by 30% of normal; 3 patients improved by 15% of normal; and 1 patient improved by 5% of normal. In the 8 patients who were greater than 50 years old, 3 patients went into full remission for 3 months or longer, 2 patients went into full remission for 6 weeks or longer, and 3 patients improved their baseline functioning by greater than 50% of normal. Although there were fewer patients (8 vs. 17) greater than 50 years old, equal numbers (5 patients in each group) went into remission, and all patients had significant improvement.

Length of illness and gender also did not affect remission rates or clinical improvement. Sixteen patients (64%) were sick for 10–20 years or longer, and 8/16 (50%) were in remission, with 8/16 (50%) showing significant clinical improvement. Among 5 patients who had been ill for more than 20 years, 1 was in full remission (3 months or longer), 2 were in remission for 6 weeks, one patient had improved by 30%, and another patient had improved by 15%; Eleven patients were sick between 10 and 20 years old, and 5 went into full remission; 4 were improved by greater than 50% of normal; and 2 patients were improved by 15% of normal. Eight patients were sick between 5 and 9 years, and one was in full remission; one was in remission for 6 weeks; 3 improved by 50% or greater; 2 improved between 30 and 45%; and one improved by 5%; and finally, one patient was sick for 0–4 years and was in full remission.

Gender also had no effect on clinical improvement or rates of remission. Although there were more men (15) than women (10) who took DDDCT and HDDCT, equal numbers of men and women (5 men, 5 women) were in remission. Among those who did not go into full remission, 60% of women (3/5) and 53.3% of men (8/15) also improved by 30% or greater, with 2 women and 2 men both having improvements ranging from 5–15%. In a recent study by Johnson et al., using self-reported clinical data from 2170 patients in the MyLymeData patient registry [157], the authors found no differences in antibiotic treatment response or side effects between women and men. We found similar results and agree that biological sex should be integrated into Lyme disease research as a distinct variable since male and female hormones can be affected by *Borrelia burgdorferi* [50,158,159]. This is especially important for women who may have hormonal flare-ups of symptoms during their menses in Lyme disease, as seen in patient number 10. She was asymptomatic for 2 months after DDDCT and several 4-day pulses of HDDCT and relapsed during her menstrual cycle, implying, from a clinical perspective, an ongoing low-grade persistent infection.

Examining the clinical symptomatology and difference in response rates of those patients who had evidence of exposure to several *Babesia* spp. and several *Bartonella* spp. simultaneously, these patients were some of the sickest and most resistant patients. Four out of five of these patients (80%) with multiple species of *Babesia* and *Bartonella* (80%) were *Bartonella* FISH-positive. One patient who was *Bartonella* FISH+ and had *Bartonella* striae suffered from psychosis with auditory hallucinations, and clinical characteristics shared among the other patients included severe neuropsychiatric symptoms (depression, anxiety) as well as resistant neuropathy (peripheral neuropathy, autonomic neuropathy). Patient 11 had chronic inflammatory demyelinating polyneuropathy, i.e., CIDP on IVIG; patient 21 had small fiber neuropathy and was on IVIG; and patient 23 also had CIDP and small fiber neuropathy. Patients 11, 17, 21, 23, and 24 had resistant neuropathic symptoms of tingling, numbness, burning, or stabbing sensations, and patients 11, 21, and 23 had severe POTS/dysautonomia, a form of autonomic neuropathy. The species of *Babesia* and *Bartonella* that were found in these 5 patients (see Table 4) included evidence of exposure to *B. microti*, *B. duncani*, *B. divergens*, *B. odocoilei*, and multiple species of *Bartonella*, including *B. henselae*, *B. quintana*, *B. elizabethae*, *B. vinsonii* subspp., *B. koehlerae*, and *B. bacilliformis*. Patients 11 and 17 had evidence of 2 species of *Babesia* (*B. microti*, *B. duncani*, and/or *B. odocoilei*), and patients 15 and 23 had evidence of 3 species of *Babesia* (*B. microti*, *B*. *duncani*, and/or *B. odocoilei*, and/or *B. divergens*). Patients 11 and 21 had evidence of 2 spp. of *Bartonella* (Bh, Be, or Bq), patients 12 and 14 had evidence of 3 spp. of *Bartonella* (Bh, Bq, Be, and Bv), and patients 15 and 23 had evidence of 4 spp. of *Bartonella* (Bh, Bq, and/or Be and Bv, or Bk and BBac). Patients who simultaneously had two or more species of *Babesia* and *Bartonella* oftentimes required psychiatric medication to help control their depression/anxiety and medication (gabapentin (Neurontin), amitriptyline (Elavil), pregabalin (Lyrica), and/or duloxetine (Cymbalta)) to control neuropathic symptoms [160,161,162,163]. The autonomic neuropathy with POTS in patient 21 was so severe that despite multiple medications used to treat POTS, including fludrocortisone (Florinef) [164], midodrine [165], droxidopa [166], Beta blockers [167], and ivabradine (Corlanor) [168], she was still unable to remain in an upright position for more than 10–15 s without developing disabling tachycardia (heart rates greater than 140 BPM) and severe dizziness with pre-syncope. This patient had two species of *Bartonella* (Bh and Bq) with elevated VEGF and low positive *tularemia* and *Brucella* titers, which both can cross-react with other intracellular pathogens [169,170].

Despite changes in a number of laboratory values during the course of treatment described here, all patient values returned to normal at the end of treatment (except for patient 24, who had a history of low platelet levels in the past). Laboratory values with a CBC, CMP (electrolytes, liver, and kidney function), and methemoglobin levels were monitored before, during, and after DDDCT and HDDCT. For some patients, there were temporary changes in white cell counts (4 episodes of leucopenia, 1 episode of leukocytosis), platelets (3 patients had mild thrombocytopenia), transient elevation in myelocytes and metamyelocytes (4–5 patients), regular decreases in hemoglobin (Hb) and hematocrit (Hct) and expected rises in the mean corpuscular volume (MCV) while on dapsone, due to the drug’s effect as a folic acid inhibitor, affecting bone marrow production [110]. There were also transient mild liver function abnormalities (5–7 patients with changes in AST and ALT), with 12 patients having transient elevations in bilirubin [171]. Methemoglobin levels rose in most patients due to dapsone’s potent oxidative effects [172]. Among 23 patients where methemoglobin levels were regularly measured on DDDCT and HDDCT, 65.2% (15/23) were less than 5%, 26% (6/23) were between 5 and 10%, and 8.6% (2/23) were between 11–20%, as long as patients took the maximum dose of methylene blue (300 mg PO BID) with other antioxidants (glutathione, Vit C, Vit E, NADH) [173]. See Protocols 1 and 2 and Table 1, Table 2 and Table 3 for the full protocol. Most patients had no symptoms of methemoglobinemia when the level was less than 5%, minimal symptoms between 5 and 10% (slight blue hands and lips, some dyspnea), and the two patients who had levels greater than 10% (pt 7, 15) tolerated the transient elevations without difficulty [173]. All abnormal values returned to normal over time.

The details of the changes in laboratory parameters were as follows: White cell counts remained WNL for 84% (21/25) of patients (WBC normal range 3500–11,000 µL), with a mean WBC count of 5.57 per µL (median 4.75 µL). Four patients (16%) had temporary episodes of leucopenia (minimum 2.36 µL) and one patient had a temporary episode of leukocytosis (maximum 18.5 µL). All WBC values returned within normal limits post therapy. Hemoglobin (Hb) values (normal Hb level for males: 14–18 g/dL; females: 12–16 g/dL) [174] were monitored for total changes in values over time, pre- and post-dapsone combination therapy. The mean drop in Hb on dapsone was 3.49 g/dL among 24 patients measured (median 3.35 g/dL; range 4.7 g/dL; minimum 1.6 g/dL; maximum 6.3 g/dL; SD = 1.364), and the drops in Hb varied based on dapsone and folic acid dosage. The higher the dose of dapsone, the more folic acid was needed to help prevent dapsone-induced anemia (see Table 1, Table 2 and Table 3). All hemoglobin values returned to baseline levels up to 2 months post-dapsone combination therapy. Hematocrit (Hct) values (normal Hct for men: 40 to 54%; women: 36 to 48%) [174] were also measured pre- and post-dapsone. The mean decrease in HCT was 10.21% (median 9.3%; range 14.1, minimum 4.8%, maximum 18.9%; SD = 3.95). All hematocrits returned WNL within several months post-dapsone therapy, as long as patients remained on high-dose folic acid and addressed any iron or vitamin deficiencies (B12) [175]. The mean corpuscular volume (MCV), which measures the average size and volume of red cells, temporarily increased in all participants on dapsone (i.e., they developed macrocytic anemia) due to dapsone’s effect as a folic acid antagonist [176]. Among 18 patients where the MCV was measured before and after DDS (normal MCV: between 80 and 100 fL), the mean increase in MCV was 13.75 fL (median 12.15; range 27.4, minimum 2.6 fL, maximum 30 fL; SD = 7.80). Mean corpuscular volume decreased back to normal in all patients after several months post-DDDCT and HDDCT as long as patients remained on high-dose folic acid and addressed any overlapping B12 or iron deficiencies. Platelets remained WNL for 22/25 patients (88%) during and post-dapsone combination therapy (normal range 135–400 k). Two patients (8%) had mild drops in platelet counts (98 k and 126 k), which returned to normal over time. One patient with a history of HELLP syndrome (Hemolysis, Elevated Liver enzymes, Low Platelets) [177] had a platelet count of 62 k post HDDCT, which has been slowly returning to normal post therapy on high dose folic acid and B12 as she suffers from ongoing inflammation [178] i.e., sedimentation rate (ESR) 55 (normal range for a female <50 years old: ≤20 mm/h) [179], with an elevated CRP 8.2 (normal range < 0.3 mg/dL) [180] as well as chronic gastritis and low B12 levels. There was no evidence of any bleeding post-dapsone combination therapy, as no platelet counts decreased below 50 k [181]. The last hematological markers we followed in patients’ CBCs were basophil counts and immature neutrophils (myelocytes and metamyelocyte counts) [182]. Among 25 patients who took DDDCT and HDDCT, 2 patients (8%) had temporary increases in basophil counts, 4 patients (16%) had temporary elevations in metamyelocytes (normal range: 0; mean increase 1.125%; median 1%; range 0.5, minimum 1%, maximum 1.5%; SD = 0.25), and 5 patients (20%) had temporary elevations in myelocytes (normal range: 0; mean increase 2.58%; median 2%; range 4.9, 1–5.9%; SD = 1.94) [183], which are hematological abnormalities that can be seen with bacterial infections [184]. Three of the four patients (75%) with elevated myelocytes (patients 3, 6, and 9; see Table 2 below) went into remission, as did two of the four patients (50%; patients 3, 9) with elevated metamyelocytes.

The last laboratory parameters we monitored before, during, and after DDDCT and HDDCT were liver functions, including aspartate transaminase (AST; normal range 5–30 IU/L), alanine transaminase (ALT; normal range 4–36 IU/L), alkaline phosphatase (AlkPt; normal range 44–147 IU/L), and total bilirubin (T. bili; normal range 0.1–1.2 mg/dL) [185,186]. AST levels were normal in 19/24 patients measured (79.2%); however, temporary borderline increases in AST were seen in 5 patients (mean increase 9.4 IU/L; median 9; range 9, minimum 4-maximum 13; SD = 3.51), and temporary mild increases in ALT levels were seen in 29.2% of patients (7/24; mean increase 24.86 IU/L; median 26; range 51; minimum 2, maximum 53; SD = 19.44). Both transaminases returned to WNL post-dapsone combination therapy. Eighty-eight percent (21/24 patients) had normal alkaline phosphatase levels (including normal increases during a growth spurt); however, 3/24 patients (12%) had multiple low alkaline phosphatase levels (patients 5, 9, and 10) before or during dapsone combination therapy. All 3 patients were in remission at the end of therapy and had a prior history of low B12 levels, which can be associated with low alkaline phosphatase [187]. Finally, 12/24 patients (50%) had normal total bilirubin levels, although several patients had mild elevations in total bilirubin prior to dapsone combination therapy secondary to Gilbert’s syndrome [188] and one patient had elevated liver functions secondary to Lyme disease that came down with doxycycline [189]. Among 12 patients with elevated total bilirubin, the mean total increase was 0.99 mg/dL (median 0.6; range 2.9, minimum 0.3, maximum 3.2 mg/dL; SD = 0.98), and all elevations in total bilirubin returned to normal post-DDDCT and HDDCT. The DDDCT and HDDCT protocols were found to be safe if patients strictly followed the typed instructions, took the recommended doses of high-dose folic acid, had regular blood tests on DDDCT (weekly) and bi-weekly blood tests on HDDCT, and had a list of symptoms that required contacting the medical office. Table 4 (below) lists the clinical results of all 25 patients with CLD/PTLDS, associated co-infections, and MSIDS variables who took DDDCT and HDDCT.

**Table 4:** Clinical Results of 25 Patients with CLD/PTLDS and Associated Co-infections, Post DDDCT and HDDCT.

Patient	M/F	Age	# Courses of DDDCT	# Courses of HDDCT × 4 Days	# Courses of HDDCT × 5–7 Days	Bart FISH +	Bart + Spp., Striae VEGF	Bab FISH +	Bab + spp.	Full Remission # Months	>30% Impr	21–30% Impr	11–20% Impr	10–19% Impr	0–9% Impr	No C Hange	MSIDS Variables Potentially Affecting Treatment Outcome
1	F	18	1	0	1 (6 d)	Yes	Bh	No	N/A	>3 mo	N/A	N/A	N/A	N/A	N/A	N/A	CVID, POTS, Mold
2	F	23	1	0	1 (5 d)	No	Bq	No	N/A	>8 mo	N/A	N/A	N/A	N/A	N/A	N/A	MCAS Leaky Gut
3	M	54	1	0	2 (5 d)	Yes	? spp	No	N/A	>4 mo	N/A	N/A	N/A	N/A	N/A	N/A	BMD, TBRF Mitochondr EM+
4	F	35	1	5	1 (7 d)	No	Yes, Bv, striae	No	Bd	>3 mo	N/A	N/A	N/A	N/A	N/A	N/A	Bd, Anapl, COVID, HSV, Mold, Phase III adrenals, PTSD
5	M	41	1	1	0	No	No	No	No	>6 mo	N/A	N/A	N/A	N/A	N/A	N/A	EM + 21 yrs prior, Insomnia, COVID, Hypoglyc
6	M	59	1	1	0	No	No	No	Bm	>11 mo	N/A	N/A	N/A	N/A	N/A	N/A	Phase II adrenals, COVID
7	F	51	1	1	0	No	No	No	Bm	>22 mo	N/A	N/A	N/A	N/A	N/A	N/A	COVID, Mold, Vasculitis
8	M	63	1	0	1 (6 d)	No	Yes, Be	No	N/A	1 mo	N/A	N/A	N/A	N/A	N/A	N/A	COVID, EBV PCR + Mold, Low T, CVID, Sick × 22 y, Poss EM
9	M	53	3	0	1 (6 d)	No	Yes, Be	No	?	1 mo	N/A	N/A	N/A	N/A	N/A	N/A	TBRF, Mycopl PCR +, OSA, Phase II Adrenal, Inflamm, sick for 31 y
10	F	22	1	3 Bart pseudo seizures stopped	1 (6 d)	Yes	Yes, Bq	No	N/A	2 mo, relapsed during menses × sev d, then felt well	N/A	N/A	N/A	N/A	N/A	N/A	Low GSH, Phase III Adrenal, POTS
11	F	31	0 Did low dose DDS for mo’s + 100 mg for 2 week	0	1 (4½–5 days)	Yes	Yes Bh, Be	No	BmBd	No	Y, >60%	N/A	N/A	N/A	N/A	N/A	Bd 1:640 +, Bm 1:160 + Phase III adrenal, severe PTSD, Candida, POTS, CVID
12	M	22	1	1	0	No	Yes, Bh, Be, Bv	No	Bm Bd	No	Y, 50%	N/A	N/A	N/A	N/A	N/A	Bm, Bd, Phase III adrenal
13	M	45	1	2	0	No	Yes, Bh, Striae	Yes	BmBd	No? (Cognitive symptoms lingering, but OSA)	Y, >70%	N/A	N/A	N/A	N/A	N/A	OSA, Food Sensitivity, Insulin R, Low T, NASH
14	F	60	2	0	1 (6–7 days) Best protocol in 13 yrs	No	Yes, Bh, Be, Bv, ↑ VEGF	No	Bm	No	Y, >60%	N/A	N/A	N/A	N/A	N/A	Bm 1:320 + Mold, PTSD, Phase II Adrenal, POTS, Bart granuloma ↓ size post HDDCT
15	M	33	1	0	1 (7 days)	Yes	Yes, Bh, Bq, Be, Bv	Yes	Bm Bd Bo	No	Y 60%	N/A	N/A	N/A	N/A	N/A	Mold, COVID, EBV PCR +. Insomnia, Detox
16	F	52	1	0	1 (6 days)	No	Yes, Bart spp. + ↑ VEGF	No	No	No	Y >60%	N/A	N/A	N/A	N/A	N/A	Anaplasma PCR +, COVID, Phase III adrenal, Detox
17	M	48	1	1 All Sx ↓ except PNP	0	Yes	Bh, Bq, Bv	Yes	Bd Bo	No	Y >50%	N/A	N/A	N/A	N/A	N/A	Bb, Bab, Bart FISH + PTSD, Microbiome Abn, Insomnia
18	M	64	1	2	0	Yes	Bq Bv	Yes	?	Physical sx > 18 mo in remission; Cognitive relapse + ? secondary TBD	N/A	N/A	N/A	N/A	N/A	N/A	Elevated Hg blood, Detox, Moderate OSA, Phase 3 Adrenal, hypoglyc, Low B12
19	M	32	1	0 Did > 1 yr. 100 mg DDSCT	1 (6 days) but no M.B., due to psych meds.	Yes	Bart genus +, Striae	No	No	No But ↓ psychosis↓ hallucin’s Post HDDCT	Y >30%	N/A	N/A	N/A	N/A	N/A	PTSD, Detox, Mitochond, Insomnia, Vit Def
20	M	41	1 Bart Striae ↑ 1 year later	1	0	No	Bh, Striae, ↑ VEGF	No	Bm	No But most Sx gone × 21 mo’s, (f) at 95% N except sl cognitive dysfunction	Y >45%	N/A	N/A	N/A	N/A	N/A	COVID, Heavy metals, Phase II adrenal, CVA × 2, Hx PFO
21	F	19	3	3 Last course w MB 300 BID, ↑ improv	0	No	Bh, Bq, ↑ VEGF	No	Bd	No, but for the 1st time in years, had sustained improv, including paralysis	Y > 15%	N/A	N/A	N/A	N/A	N/A	Babesia ++ Severe POTS, EDS, Phase III adrenal, AI IVIG/SQIG, Leaky gut, MCAS, Immune dys(f)
22	M	34	1	4	0	Yes	Bart genus	No	Bd	No	Y 30%	N/A	N/A	N/A	N/A	N/A	Mold +++ Babesia ++ CVID (not yet on IVIG), low T, NASH, insomnia
23	M	33	1	0	2 (7 days)	Yes	Bh, Bq, Bk, Bbac Striae	No	Bm Bd, B div	No	N/A	N/A	Y 15%	N/A	N/A	N/A	Bab ++ Mold +++ POTS MCAS CIDP COVID w/EBV PCR+ S/P EM
24	F	45	2	3 Lower dose MB used	1 (6 d) Dapsone helped ↓ Morgellon’s lesions	Yes	Bh	Yes	?	No	No	No	No	Y, 15%	N/A	N/A	Morgellons CVID Hypopit:, Phase III adrenal, hypothy, ↓ sex hormones, PTSD, insomnia +++ POTS, Mitochondr dysfunction Inflamm ↑ Vit/min def AI dx with HELLP Syndrome
25	M	32	1 (only did 2 weeks)	1 Lower dose MB used (150 mg BID)	0	No	No	Yes	Bd	No	No	No	No	No	Y, 5%	N/A	Mold Babesia ++ PTSD Phase II Adrenal. States (f) at 90% N

Abbreviations: Male (M); Female (F); Double Dose Dapsone Combination Therapy (DDDCT). High Dose Dapsone Combination Therapy (HDDCT); Anaplasma (Anapl); *Bartonella* fluorescent in situ hybridization. (Bart FISH); *Bartonella* species (Bart spp.); *Bartonella henselae* (Bh); *Bartonella quintana* (Bq); *Bartonella elizabethae* (Be); *Bartonella vinsonii* subspp. (Bv); *Bartonella koehlerae* (Bk); *Bartonella bacilliformis* (Bbac); vascular endothelial growth factor (VEGF); *Babesia* fluorescent in situ hybridization (Bab FISH); *Babesia* species (Bab spp.); *Babesia microti* (Bm). *Babesia duncani* (Bd); *Babesia odocoilei* (Bo); *Babesia divergens* (Bdiv); Autoimmune (AI); *Borrelia Miyamotoi* Disease (BMD); Coronavirus-19 (COVID); Chronic Variable Immune Deficiency (CVID); Chronic Inflammatory Demyelinating Polyneuropathy (CIDP); Cerebrovascular Accident (CVA); Detoxification problems (Detox); Dysfunction (Dys(f); Epstein Barr Virus (EBV); Erythema migrans rash (EM); Functioning (f); Glutathione Deficiency (GSH); Hemolysis and Elevated Liver Functions with Low Platelets (HELLP Syndrome); Herpes viruses (HSV). Hypoglycemia (hypoglyc); Hypopituitarism (hypopit); hypothyroidism (hypothy); Improvement (Impr). Inflammation (Inflamm); Insulin resistance (Insulin R); Intravenous Immunoglobulin (IVIG); Low testosterone (low T). Mast Cell Activation Syndrome (MCAS); Mercury (Hg); Microbiome abnormalities (Microbiome abn). Mitochondrial Dysfunction (Mitocondr); Non-alcoholic Steatohepatitis (NASH); Obstructive Sleep Apnea (OSA); Polymerase Chain Reaction (PCR); Postural Orthostatic Tachycardia Syndrome (POTS); Posttraumatic Stress Disorder (PTSD); Subcutaneous Immunoglobulin therapy (SQIG): Tickborne Relapsing Fever (TBRF); Vitamin Deficiency (Vit Def). Symbols: Number (#); Unidentified (?); Increased (↑); Decreased (↓); moderate intensity (++); severe intensity (+++).

## 4. Discussion

Lyme disease has reached epidemic proportions in the United States and worldwide, with an estimated global Bb seroprevalence of 14.5% [2]. Although 75–80% of patients improve with short-term antibiotic therapy early in the course of LB, at least 20–25% may go on to develop Chronic Lyme disease/PTLDS, a multisystem disabling chronic fatiguing, musculoskeletal, and cardiologic illness with neuropsychiatric symptoms [190]. The etiology of CLD/PTLDS has been debated in the scientific literature for more than 40 years. In three prior studies involving 265 patients with CLD/PTLDS [51,102,103], the vast majority of these patients had evidence of multiple active co-infections, including but not limited to *Babesia* and *Bartonella*, with some patients reactivating underlying viral infections (HHV-6, EBV). Our cohort of CLD patients also had other overlapping sources of inflammation on the 16-point MSIDS map contributing to their persistent illness, including environmental toxins (heavy metals, mold), microbiome abnormalities, leaky gut with food sensitivities/mast cell activation syndrome (MCAS), sleep disorders, and vitamin and mineral deficiencies [50,102,103]. These multiple sources of inflammation oftentimes resulted in downstream effects including hormonal dysregulation (adrenal dysfunction, low testosterone), pain syndromes (chronic muscle and joint pain, resistant neuropathy), neurological/psychological dysfunction (depression, anxiety, occasionally OCD, bipolar disorder, and psychosis), autoimmune reactions (positive ANAs, rheumatoid factors, anti-ganglioside, anti-myelin antibodies, and anti-thyroid antibodies), autonomic neuropathy (POTS/dysautonomia, gastroparesis), liver function abnormalities (elevations in transaminases) as well as mitochondrial dysfunction [50,70,71]. We found the same abnormalities contributing to chronic illness in this current cohort of 25 patients (see Table 4), where active *Babesia* and active *Bartonella* played a major contributing role in driving chronic symptomatology. Sixteen out of 25 patients (64%) with CLD/PTLDS had exposure to *Babesia* spp., and 21/25 patients (84%) tested positive for one or more *Bartonella* species. The 3 patients with CLD/PTLDS (patients 5, 6, and 7) who went into long-term remission with 8 weeks of DDDCT and one 4-day pulse of HDDCT had no evidence of active *Babesia* or *Bartonella*, despite an LOI between 11 and 21 years. None of the 6 patients who had active *Babesia* by FISH went into full remission, requiring multiple rotations of anti-malarial medications, and those patients who had evidence of exposure to multiple species of *Babesia* and *Bartonella* were some of the sickest patients with resistant neuropsychiatric symptoms and peripheral/autonomic neuropathy (POTS). In those Lyme and *Bartonella* patients who were FISH+ (and/or had an elevated VEGF and/or striae with granulomas) with a history of *Babesia* exposure (FISH negative), a longer course of HDDCT (5–7 days) was required post-8 weeks of DDDCT to achieve full remission. Seven out of 23 patients (30.5%) with CLD/PTLDS with *Bartonella* and *Babesia* who completed 8 weeks of DDDCT followed by a 6 to 7 day pulse of HDDCT remained in remission for a period of 3–9 months, and 3/23 patients (13%) recently completed the protocol and so far have been in remission for at least 1 ½ months. Although 100% of all 25 patients had an improvement in underlying symptomatology with DDDCT and HDDCT, and 43.5% of patients were in remission at the time of submission of this publication, overlapping MSIDS factors interfered with many patients who did not go into full remission. These factors primarily included inflammation/autoimmune phenomena (80%), due to not only chronic infections but also environmental toxins with mold and heavy metals (88%), immune dysfunction with CVID and CIDP (36%), hormonal abnormalities with low adrenal function, low testosterone (low T) (80%), leaky gut/food sensitivities/microbiome abnormalities/MCAS (68%), hypoglycemia/candida (up to 52%), vitamin and mineral deficiencies (88%), detoxification abnormalities including low glutathione (28%), liver abnormalities, including non-alcoholic steatohepatitis (NASH) (56%), resistant insomnia (68%), POTS/dysautonomia (32%), and severe neuropsychiatric symptoms (56%). Establishing a broad differential diagnosis among those suffering from CLD/PTLDS and screening with the 16-point MSIDS map is essential from our perspective, since many of these MSIDS factors can cause resistant fatigue, pain, cognitive difficulties, insomnia, and neuropsychiatric symptoms resembling chronic Lyme and tick-borne infections, yet their treatment and resolution do not involve antibiotic therapy.

Long COVID also has overlapping symptoms resembling CLD/PTLDS, including chronic fatigue, muscle/joint and nerve pain, insomnia, “brain fog” with cognitive difficulties, neuropsychiatric problems, chest pain, shortness of breath, palpitations, and dizziness secondary to POTS [191]. One way to help differentiate long COVID from CLD is that anosmia, dysgeusia, cardiovascular, and pulmonary complications are more common and constant in long COVID [192,193], whereas, with CLD, symptoms tend to come and go with good and bad days, patients oftentimes have an improvement or worsening of symptoms with antibiotics (Herxheimer reactions) [130], and one of the hallmark symptoms of both acute and chronic Lyme disease is migratory muscle pain, joint pain, and/or nerve pain (neuropathy) [194]. A validated screening questionnaire for Lyme disease is available for establishing a differential diagnosis [194]. Many of the patients in this study had migratory pain, and many had been exposed to both illnesses. Fifty-two percent of patients in this paper had COVID-19 during the course of their dapsone therapy, and the viral overlap worsened their underlying tick-borne symptoms post-COVID despite treatment with Nirmatrelvir/Ritonavir (Paxlovid) [195]. Two patients (patients 15, 23) reactivated EBV post-COVID-19, and several patients had temporary Herxheimer reactions after COVID vaccination, implying an overstimulated immune system. In general, COVID vaccination was well tolerated, and there were no long-term effects in our population of patients with CLD and associated co-infections [196]. Patient 4 had a history of CLD, *Babesia duncani*, and *Bartonella vinsonii* subspp., had been vaccinated and boosted several times against COVID-19, and contracted COVID-19 seven times during the course of her illness due to her job, which required regular travel across continents. Despite 7 episodes of COVID-19, one course of DDDCT, and 5 courses of 4-day pulses of HDDCT, her health significantly improved, with each pulse of HDDCT removing a resistant symptom. It was not until she did one 7-day pulse of HDDCT with 300 mg of MB for Lyme and *Bartonella* that she went into full remission for 3 months or longer.

Dapsone (DDS) alone or in combination with other antibiotics (doxycycline) has been proposed as a potential treatment for those with COVID-19 due to the drug’s combined effects in blocking inflammatory storms and suppressing the production of cytokine signatures including IL1α, IL8, IL1β, IL6, and IL8 and tumor necrosis factor-α [197,198]. DDS inhibits neutrophil myeloperoxidase, inflammation, and neutrophil chemotaxis, as well as the expression of inflammatory signaling pathways and the generation of reactive oxygen species (ROS), known complications of COVID-19 [199]. The positive clinical effects of DDS and COVID-19 were observed in a case series of 22 patients and 22 control subjects who rapidly recovered from Acute Respiratory Distress Syndrome (ARDS) within 24 h of taking dapsone [200]. The mortality rates were 0% with dapsone administered as a standard COVID-19 treatment at the onset of ARDS and 40% mortality without dapsone as a standard COVID-19 treatment, respectively [200]. A follow-up study evaluating dapsone’s effect on COVID-19 showed a lower risk of death and discharge to long-term acute care (LTAC) and a higher chance of discharge home among patients receiving dapsone at a dose of at least 100 mg PO QD or BID (the dose used in DDDCT) compared to those receiving the usual standard of care [201]. Side effects of DDS in these hospitalized patients were minimized using cimetidine 400 mg three times daily to diminish dapsone-related methemoglobinemia [202], with no episodes of hemolytic anemia [201]. Dapsone’s anti-inflammatory and neuroprotective effects were also statistically proven in a randomized, controlled trial of 8680 leprosy patients followed over 15 years (*p*-value < 0.00001), where DDS decreased the incidence of Alzheimer’s disease exacerbation [203]. The positive effect of dapsone was felt to be due to its anti-inflammatory effect as a neuroinflammasome competitor and cyclic-GMP-AMP synthase (cGAS/STING) pathway inhibitor in the central nervous system (CNS), lowering inflammation [203]. Although *Borrelia burgdorferi* has been found in the CNS in patients with Alzheimer’s disease [204,205,206,207,208], none of the patients in that study who took dapsone were examined for Lyme disease or overlapping MSIDS variables that could potentially affect cognition.

Seven of the factors found on the 16-point MSIDS factors in those suffering from CLD/PTLDS due to ongoing inflammation and downstream effects have been reported in the long COVID-19 syndrome, i.e., viral persistence (coronavirus), viral reactivation (EBV), autoimmunity, mitochondrial dysfunction, POTS/dysautonomia, dysbiosis of the gastrointestinal tract, and MCAS [209,210,211,212,213,214,215]. The common denominator in both chronic fatiguing musculoskeletal, cardiovascular, and neuropsychiatric illnesses, i.e., CLD/PTLDS and long COVID, are the 3 I’s: infection, inflammation, and immune dysfunction. These are hypothesized to be primarily due to viral insults in COVID, i.e., the persistence of the coronavirus and resultant hyperproduction of proinflammatory cytokines with downstream effects and immune dysregulation, which are proposed to form the basis of the long COVID syndrome [216]. In Lyme disease, a similar clinical syndrome has been proposed to be due to the persistence of *Borrelia* after standard courses of antibiotics, increasing inflammation [23]. The scientific literature has established that Lyme spirochetes may form drug-tolerant *Borrelia* “persisters” in biofilms [72,75,90,217], hypothesizing that patients with CLD/PTLDS may remain ill after standard treatment with antibiotics due to these antimicrobial-tolerant persisters [218] in biofilms [73,91,219], increasing production of proinflammatory cytokines [77].

There are multiple scientific studies showing that *Borrelia* can persist after standard courses of antibiotics [220,221,222], including inside the joints [223,224], ligaments [225], fibroblasts [46,84], intracellular compartment [44], endothelial cells [82], skin [226,227], eyes [228], and central nervous system [229,230], with recent studies implying a role for microbial biofilms in Lyme neuroborreliosis [91]. Persistence of *Borrelia* has similarly been proven in the animal model [231], in mice [20], dogs [21,232], horses [233], and macaques [22,234], as well as humans [23,24,51,235]; however, only two repurposed medications that address Lyme persisters have undergone clinical evaluations to date: disulfiram (DSF) [96,97] and dapsone [50,51,102,103,104]. Both are medications that have been shown to have an effect on *Borrelia*. Although 1–2 courses of high-dose DSF have been shown to have some clinical efficacy in patients with CLD and associated co-infections, resulting in remission rates of up to 36.4% with 6–18 months of use [97], disulfiram as a single drug and/or in combinations against stationary-phase *B. burgdorferi* persisters was shown to lack efficacy (survival rate (SR) 46.3%) [236]. Dapsone (DDS), a sulfa drug, was evaluated by Johns Hopkins University researchers and compared to DSF. It was found to be effective against *Borrelia burgdorferi* stationary phase persisters in biofilms, both as a single drug and in combination [108]. They concluded regarding dapsone that “sulfa drugs combined with other antibiotics were more active than their respective single drugs” and that four-drug combinations were more active than three-drug combinations. “The four-drug combinations dapsone + minocycline + cefuroxime + azithromycin and dapsone + minocycline + cefuroxime + rifampin showed the best activity against stationary phase *B. burgdorferi* in these sulfa drug combinations” [108]. Dr. Eva Sapi and her research team at the University of New Haven confirmed these findings on dapsone’s efficacy against *Borrelia* persisters in culture after evaluating dapsone as a single agent or in combination with tetracycline, rifampin, and azithromycin [95]. The conclusion of that study was that dapsone, as a single agent, was effective against *Borrelia* persisters; however, the efficacy against the persister/biofilm forms of Bb increased as antibiotic combinations were added. Dapsone in combination with doxycycline (2-drug combo) was more effective than dapsone alone, and dapsone + doxycycline + rifampin (3-drug combo) was more effective than the 2-drug combination. The most significant effect in reducing the mass and viability as well as the protective mucopolysaccharide layers of *B. burgdorferi* biofilm was, however, using the 4-drug combination of dapsone + doxycycline + rifampin + azithromycin [95]. The study by Sapi et al. confirmed the culture findings found by Feng et al. at Johns Hopkins University on the superior efficacy of dapsone against *Borrelia* persisters using multiple antibiotic combinations [108]. To date, there has been one animal study by Embers et al. (pre-print) proving dapsone’s efficacy in Lyme disease in a mouse model [237], which confirmed the superior efficacy of combination antibiotic therapy vs. monotherapy in the treatment of borreliosis. The findings of that study were that “none of the monotherapies eradicated persistent Bb; however, 4 dual combinations, including dapsone + rifampicin, dapsone + clofazimine, and 3 triple combinations (including dapsone + rifampicin + clofazimine), eradicated persistent Bb infections” [237].

The three-drug combination of a tetracycline, rifampin, and dapsone (100 mg BID), i.e., DDDCT, was shown to have clinical efficacy in the treatment of CLD/PTLDS, demonstrating tick-borne symptom improvements in 98% of patients, with 45% remaining in remission for 1 year or longer if they didn’t have active *Bartonella* [102]. In that study, none of the patients who were *Bartonella* FISH positive (seven patients) or PCR/biopsy positive (two patients) using lower dose dapsone went into remission [102]. Clinical efficacy for CLD/PTLDS was again demonstrated using the same three-drug combination using higher-dose pulse dapsone combination therapy (200 mg PO BID × 4 days) after 8 weeks of DDDCT, with lower doses of methylene blue (100 mg BID) added to the protocol. In that study, 84% of patients showed improvement in their tick-borne symptoms, and 32% had a resolution of all active Lyme symptoms post-treatment for 3 months or longer, even if there was evidence of prior active co-infections, including *Babesia* and *Bartonella*. The aim of this study was to examine the effect of higher-dose dapsone pulses in the treatment of resistant Lyme and associated co-infections as well as the effect of methylene blue (MB). In that study, using a 4-day HDDCT pulse with 100 mg BID of MB had some efficacy against *Babesia* and *Bartonella* but was insufficient to put most patients with active co-infections in full remission. “Among 11 patients who were *Babesia* FISH positive, three (27%) remained in remission, six improved (55%), and two (18%) had no change in sweats, chills, flushing, or dyspnea. “Among 19 patients (76%) with a history of *Bartonella* exposure, 15/19 improved (79%), and four patients (21%) had no change in symptoms” [103].

Our present protocol was the first dapsone combination therapy to regularly use a 4-drug combination of dapsone with a tetracycline (minocycline or doxycycline), rifampin (or occasionally rifabutin), and azithromycin. A 5th antibiotic, pyrazinamide, i.e., PZA (or rarely macrodantin if there was an intolerance to PZA), was also added in resistant cases, using longer courses of high-dose dapsone (5–7 days vs. 4-day regimens) with higher doses of methylene blue (up to 300 mg PO BID) for the treatment of CLD/PTLDS and associated co-infections, including *Bartonella*. Research in the past several years has shown that *Bartonella*, such as *Borrelia*, can form persisters in biofilms [238,239], and that biofilm formation is a critical step in the formation of vegetative masses during *Bartonella*-mediated endocarditis, representing a potential reservoir for the persistence of these bacteria [238]. Johns Hopkins researchers used a high-throughput screen of an FDA-approved drug library against stationary phase *B. henselae* to identify more effective treatments for persistent *Bartonella* infections [98] and evaluated the effect of different drugs and drug combinations on killing stationary phase and biofilm forms of *Bartonella henselae* in vitro [101]. Their conclusion was that a 6-day combination therapy of azithromycin and MB and rifampin and MB completely eradicated all biofilm bacteria of *Bartonella*, with no viable bacteria detected after 6-day drug exposure [101]. We, therefore, implemented that combination therapy in this present high-dose dapsone pulse for 6–7 days vs. 4 days in our past studies, along with a higher dose of MB, for the treatment of resistant *Bartonella*, one of the primary co-infections interfering with the success of DDDCT and HDDCT [102,103]. Using a “bench to bedside” approach, rifampin, azithromycin, and higher dose MB were hypothesized to not only theoretically more effectively kill *Bartonella* persisters (the Hopkins study did not identify medication dosing) but would also have the advantage of helping to lower methemoglobin levels resulting from the use of higher dose dapsone [145,172], which was used in this present cohort of patients for longer periods (6–7 days vs. 4). Methylene blue has also been shown to have positive effects on mitochondrial function and efficacy in mitigating neurodegeneration [240]. Several patients with CLD and *Bartonella* who took higher doses of MB (300 mg BID) during their pulse therapies vs. lower doses (100–200 mg) noticed improved clinical results. Patient 23 noted sustained improvements in energy, muscle and joint pain, neuropathy, and muscle strength (paralyzed muscles moved for the first time in 8 years) with a higher dose of MB. Patient 24 similarly noticed for the first time sustained improvement in energy, myalgias, neuropathy, and cognitive functioning after using a 6-day HDDCT pulse with MB 300 mg PO BID. Patient 10 became 2 months symptom-free for the first time after a 4 day HDDCT pulse and 300 mg PO BID of MB, whereas lower doses of MB with 4 day pulses (100 mg BID, 200 mg BID) helped decrease symptoms of fatigue, pain, neuropathy, brain fog, and *Bartonella* “pseudo seizures” (possible complex partial seizures) for a maximum of one month before relapsing during her menstrual cycle. She is now in full remission several months after doing her first 6-day HDDCT pulse with 300 mg PO BID of MB.

We added Pyrazinamide (PZA) for the treatment of resistant *Bartonella* in the majority of our patients based on the drug’s efficacy in mycobacterium infections, another bacterial “persister.” PZA has been published to help shorten the course of mycobacterium therapy by demonstrating efficacy in latent infections, phenotypic resistance, or tolerance [241]. A case study on CLD and *Bartonella* published several years ago in a patient with Behçet’s disease, a severe autoimmune illness, demonstrated that dapsone combination therapy along with PZA had efficacy against a stealth infection with *Bartonella*, helping to relieve resistant symptoms [105]. It was therefore chosen as the fifth medication in this cohort of patients (84%) who were chronically ill and exposed to multiple species of *Bartonella*. Among those patients who had CLD and *Bartonella* and who took 8 weeks of DDDCT and a 5–7 day course of HDDCT with PZA, 7/23 patients (30.5%) remained in full remission for 3 months or longer, and 3/23 (13%) were in remission for up to 6 weeks. Due to the small sample size and lack of a similar comparison group, we cannot conclude whether PZA increased response rates to *Bartonella*. Among three patients in our cohort with a history of CLD (patients 5, 6, and 7) without evidence of *Bartonella* who did not use PZA, 100% (3/3) went into long-term remission with an 8-week course of DDDCT and one 4-day HDDCT pulse. The 4-day HDDCT pulse was only effective in inducing long-term remission in those patients without evidence of active *Babesia* or *Bartonella*. This included patient 5, who met the criteria for PTLDS after having had an EM rash 21 years prior. He had low-grade symptoms for several years after doing an 8-week course of DDDCT. He improved significantly; however, symptoms would come and go. Relapses included several 10–14 day cycles per year, with a return of joint pain, irritability, insomnia, and word recall issues that would then spontaneously resolve. It was not until doing a 4-day HDDCT pulse with 300 mg PO BID of MB three years after taking one course of DDDCT that he went into full remission for over 6 months’ time (see Protocol 2). A double-blind, controlled, randomized trial using DDDCT followed by a 4-day HDDCT pulse needs to be conducted to confirm whether this protocol will lead to long-term remission in those patients with CLD without associated co-infections, as it did in patients 5, 6, and 7.

Regarding potential side effects of the medication regimen, one patient, patient 23, was unable to take PZA due to a transient rash that developed on the medication. It was promptly resolved using H1 and H2 blockers (cetirizine, famotidine) [242]. He had 4 species of *Bartonella* (Bh, Bq, Bk, and Bbac) with striae and 3 species of *Babesia* (Bm, Bd, and Bdiv) and had been sick for over 10 years before doing dapsone combination therapy. He had an EM rash in 2008 (meeting the criteria for PTLDS) and had previously failed 7 years of antibiotics before seeing us, including IV doxycycline for two months and IV azithromycin, as well as a short course of DSF. No antibiotic protocols during the 7 years of treatment had resulted in any significant sustained improvement. He did one course of DDDCT and two seven-day courses of HDDCT and had a 15% improvement in symptoms, including decreased muscle/joint pain, neuropathy, tinnitus, and improvements in cognition. Rotations of *Babesia* medications (tafenoquine, ivermectin, Malarone, Chinese skullcap, and Japanese Knotweed) along with dapsone were required to help decrease his sweats and air hunger, classical symptoms of Babesiosis. Dapsone’s benefit in this patient was in part from its antimalarial effects, i.e., antimicrobial as well as antiprotozoal effects, making it useful as an adjunct treatment of *Babesia* [51,102,104]; anti-inflammatory features similar to non-steroidal anti-inflammatory drugs [243], with efficacy in autoimmune disease [244,245]; as well as DDSs efficacy against the persister forms of *Bb* [95] with excellent penetration in the CNS, lowering neuroinflammation [203].

Patients with multiple species of *Bartonella* and multiple species of *Babesia*, like patient 23, were the sickest individuals in this cohort with the most severe, resistant symptoms. This may be due to the overlapping effects of *Babesia* and *Bartonella* each increasing inflammatory cytokine production [29,246], as well as *Borrelia* and *Babesia* increasing inflammation, where each pathogen affects the other microbe directly or indirectly by influencing the host immune response [247]. To the best of our knowledge, this is the first study to examine in detail the role of multiple *Bartonella* species and *Babesia* species in CLD, where patients also had other overlapping sources of inflammation with downstream effects (MSIDS variables). There are presently over 36 species of *Bartonella* that have been identified, of which at least 17 have been associated with an expanding spectrum of animal and human diseases [14,36]. We found up to six different species in our patients, including *Bartonella henselae* (associated with a virulent form of cat scratch fever and culture-negative endocarditis), *Bartonella quintana* (associated with trench fever, chronic endocarditis, and bacillary angiomatosis), *Bartonella elizabethae*, *Bartonella vinsonii* subspp., and *Bartonella koehlerae* (both associated with rheumatological manifestations) [248,249], and *Bartonella bacilliformis* (associated with Carrion’s disease, Oroya fever, and Verruga peruana). Clinical manifestations of these *Bartonella* species include a broad range of eye problems (uveitis, neuroretinitis, Parinaud oculo-glandular syndrome), cardiac problems (myocarditis, endocarditis, aneurysm formation, vasculitis, relapsing bacteremia), peripheral neuropathy, central nervous system neurological disorders (Pediatric acute-onset neuropsychiatric syndrome (PANS), hallucinations, psychosis, encephalopathy, transverse myelitis, radiculitis, cerebellar ataxia), [250] rheumatological presentations, vasculitis [251], chronic lymphadenopathy, skin problems with granulomas and bacillary angiomatosis, as well as liver involvement (Peliosis Hepatis, vascular proliferation in the liver) [250]. Recently, *Bartonella* species have also been associated with breast cancer [252,253,254] and melanomas [255], a highly concerning finding warranting more research [36].

The symptoms of *Bartonella* can overlap those of CLD, including fevers, fatigue, joint and muscle pain, headaches, cognitive difficulties, eye problems, and mood disorders [250], potentially increasing the severity of other underlying tick-borne disorders. Clinical clues that *Bartonella* may be present include severe neuropsychiatric symptoms with striae [256] (patient 19), pain on the soles of the feet upon waking in the morning [250], granulomas (patient 14) [257], and severe resistant neuropathy, both peripheral neuropathy and autonomic neuropathy (patients 17, 21, 23, 24). Immune dysregulation and immune deficiency may also be clues that *Bartonella* is present. We believe that *Bartonella* infections in our study interfered with the long-term efficacy of HDDCT, as did multiple *Babesia* species, necessitating a differential diagnosis and laboratory testing for multiple species of each infectious agent. Several of these *Bartonella* species have been associated with immunosuppression. These include *B. bacilliformis*, *B. henselae*, and *B. quintana* [258]. Several of our patients with CLD and *Bartonella* had Chronic Variable Immune Deficiency (CVID), i.e., patients 1, 8, 11, 22, and 24, and patient 23, who had 4 species of *Bartonella* (Bh, Bq, Bk, BBac), had Chronic Inflammatory Demyelinating Polyneuropathy (CIDP). Considering the high incidence of immunosuppression in 5/25 patients (20%) as well as neuropathy (autonomic and peripheral) with neuropsychiatric symptoms among this cohort of patients, any patients presenting with CLD and associated co-infections with the above clinical symptomatology warrant initial laboratory testing for *Bartonella* species and immune deficiency. This evaluation should include an immunoglobulin panel (IgA, IgM, IgG, and IgE) with IgG subclasses [259], with consideration of referral to an immunologist for more detailed testing. A comprehensive search for multiple *Bartonella* species is reasonable in this clinical setting, as well as other co-infections and MSIDS variables, since not only has *Bartonella* been associated with immune deficiency [258]; therefore, has Lyme disease [50], *Anaplasma* [260], *Babesia* [261], as well as mold toxins, including gliotoxins [262]. Among 25 patients having evidence of 6 different mold toxins, 44% of our patients (11/25) had evidence of gliotoxins, as well as 28% showing evidence of ochratoxins (7/25), 44% (11/25) Trichothecenes, 32% (8/25) aflatoxins, 36% (9/25) Zearalenone, and one patient (4%) had evidence of citrinin/dihydrocitinone. Testing should therefore include a broad differential diagnosis and include not only antibody testing for *Bartonella*, which can lack adequate sensitivity [100]; however, PCR [263], real-time PCR [264,265], droplet digital PCR (ddPCR), which can amplify DNA of over 25 *Bartonella* spp. [266], as well as *Bartonella* immunoblots [267] and *Bartonella* FISH testing [268] which can check for the presence of multiple *Bartonella* species. We found *Bartonella* FISH testing (IgeneX laboratories) to be positive in 11/25 patients (44%) and helpful in proving an active *Bartonella* infection as a cause of persistent symptoms when other serological methods were negative. The vascular endothelial growth factor (VEGF) test was also useful as an indirect marker of active *Bartonella* [269].

**Figure 3:**
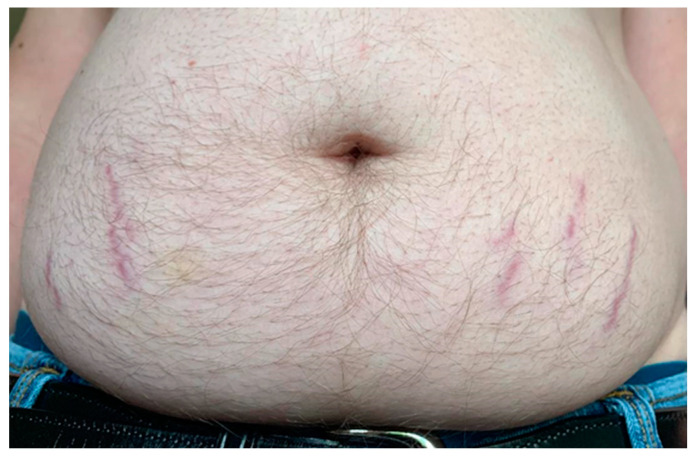
*Bartonella* striae which emerged one year post DDDCT.

*Bartonella* species are fastidious, gram-negative, vector-borne bacteria that can cause long-lasting, relapsing intraerythrocytic bacteremia and intraendothelial infections [270]. At least four *Bartonella* spp. (*B. henselae*, *B. quintana*, *B. bacilliformis*, and *B. vinsonii* subsp. *berkhoffii*) have been shown to induce VEGF-mediated vasoproliferative disease in immunocompromised or immunocompetent animals (cats, dogs) and humans [270], as *B. vinsonii* subsp. *berkhoffii*, *B. henselae*, and other *Bartonella* spp. have developed survival mechanisms that allow for low-level intravascular and intracellular persistence [270]. We found all 4 species in our patients, with 3 patients having intermittent elevated VEGF levels during the course of treatment. Patient 14 had been disabled for 26 years with a history of CLD (CDC IgM Immunoblot+), *Babesia microti* (1:320+), and *Bartonella* (*Bartonella* Western Blot+; *B. vinsonii* subspp., *B. henselae*, and *B. elizabethae*) with a history of severe fatigue, joint pain, sweats, and shaking chills (*Babesia*) with severe memory/concentration problems, depression, and panic attacks. She had been on high-dose morphine sulfate years ago before seeing us due to her severe pain, which was still not controlled by narcotics. She did two courses of DDDCT in the past several years, which improved her health and allowed her to stop all her narcotics as her pain levels decreased. She recently did one 6–7 day course of HDDCT with 300 mg of MB PO BID at the end of December 2022, several years later. After having a severe Herxheimer reaction, she improved from 25% to 87% of normal functioning, and a *Bartonella* granuloma on her hand decreased in size by approximately 80% (see Figure 2). She felt that this protocol was the most effective antibiotic regimen she had tried in 26 years, with higher doses of MB having a significant positive effect as her hand started healing as the dose of MB was gradually increased. Patient 16 had a history of CLD with migratory joint pain, an indeterminate *Bartonella* immunoblot with elevated VEGF, and a positive *Anaplasma* PCR in her blood. She suffered from bipolar disorder, chronic fatigue, memory/concentration problems, and severe joint pain with elevated markers of inflammation (rheumatoid factor, ESR, and CRP). She did one 8-week course of DDDCT in August 2022, followed by a 6-day HDDCT pulse in March 2023, and had significant improvements in arthritic joint pain, energy, sleep, and cognition. She started at 23–30% of normal and was now functioning at 80% of normal as of May 2023. Finally, patient number 20 had a history of CLD, *Babesia microti* (1:64+), an indeterminate *Bartonella* immunoblot with *Bartonella henselae* (1:256+), and elevated VEGF. His primary symptoms in 2017 included severe fatigue, myalgias, rib pain, headaches, neuropathy, lightheadedness, cognitive difficulties, and mood swings. He did one 8-week course of DDDCT in March 2019 with doxycycline, rifampin, azithromycin, and pyrazinamide, and although he improved, one year later, in April 2020, symptoms relapsed after being treated for cellulitis with Augmentin when *Bartonella* striae emerged (see Figure 3). The DDDCT protocol, although helpful, was insufficient to clear *Bartonella*, as evidenced by the emergence of classical striae one year later on antibiotics. He did one course of 4 days of HDDCT in September 2021 with a lower dose of MB (100 mg BID) and went from 50–60% normal to 95% normal functioning. He would have been counted as in remission if not for his occasional word-finding problems and sleep issues. This patient had multiple overlapping MSIDS variables affecting his resistant symptoms.

There are several unanswered questions regarding the etiology and role of *Bartonella* in CLD, which is controversial, and whether it is definitely a tick-transmitted pathogen. *Bartonella* spp. can be transmitted from mammals to humans via cat fleas, sand flies, keds, mites, and human body lice [14]. Recent evidence also suggests transmission of *Bartonella* via ticks, red ants, and spiders [36]. Further studies need to be performed, however, to evaluate the vector competence of *Bartonella* transmission by ticks, although one study in Europe with *Ixodes ricinus* ticks confirmed their ability to transmit a species of *Bartonella*, *B. birtlesii* [271]. Whether Lyme disease patients are getting *Bartonella* from ticks, which have been shown to contain different *Bartonella* spp. [272,273,274,275] or from other insect vectors is unresolved; however, recent scientific studies are demonstrating that a significant percentage of LD patients are contracting *Bartonella* [51,102,103]. In one study, 78% of the patients with chronic Lyme disease proved to be seropositive for *Bartonella henselae* [19]. In our cohort of 25 patients, 84% tested positive for one or more *Bartonella* species. These included: 11 patients with *Bartonella henselae*; 7 with *Bartonella quintana*; 6 with *Bartonella elizabethae*; 6 with *Bartonella vinsonii* subspp.; 1 with *Bartonella koehlerae*; 1 with *Bartonella bacciliformis*; and 4 with *Bartonella* spp. (genus) without specific spp. identification. The MyLymeData patient registry of about 4000 participants highlighted the large heterogeneity of treatment responses for those with CLD [276]. Based on our present population and prior published articles on dapsone combination therapy for the treatment of CLD/PTLDS and associated co-infections [50,51,102,103,104,105], it is possible that undiagnosed/untreated *Bartonella* and *Babesia*, undiagnosed/untreated MSIDS factors, as well as the large variability in treatments, including newer persister drug regimens, accounted in part for the large diversity of treatment outcomes.

*Babesia*, a malarial-type parasite found in ticks [277], which can also be transmitted by blood transfusion [278], maternal-fetal transmission [279,280], and solid organ transplantation [281], was found in 64% (16/25) of our patients. Several patients had more than one species present. Nine patients had evidence of *Babesia microti*; 10 *had Babesia duncani*; 2 *had Babesia odocoilei* (FISH); 1 *had Babesia divergens;* and 20% (5/25) had evidence of active *Babesia*, i.e., were *Babesia* FISH positive. The primary symptoms present in those with active *Babesia* included day sweats, night sweats, chills, flushing, an unexplained cough, and “air *hunger*”, which are questions 1 and 22 on the HMQ-validated Lyme questionnaire [194]. Those patients with active *Babesia* were more resistant to treatment, and none of the 6 patients (24%) who were *Babesia* FISH+ remained in full remission. Although the range of a *B. microti* lineage has been known to be expanding in the Northeastern US [282] as well as *the* Southeastern US [283], it has recently been found in Europe along with *B. divergens* and *Babesia venatorum* [284]. Patient 13 from Europe had evidence of both *B. microti* and *B. duncani*. *Babesia duncani* has also been increasing its range from the Western US to *the* Eastern US [51,285] as well as Canada [286], and should therefore be regularly included in serological studies of patients suspected of having Babesiosis. The newest piroplasm species to be discovered in *Ixodes* ticks is *B. odocoilei* [287,288,289]. *B. odocoilei* has been found in ticks from Canada to Texas [290], and recently, several human cases of *B. odocoilei* were found by Scott et al., implying that the parasite can be pathogenic in humans [291]. Since *B. odocoilei* serologically cross-reacts with *Babesia duncani* [291], it is possible that some resistant cases of Babesiosis may be due to this emerging parasite; however, more scientific data are needed before any conclusions can be reached. The *eight* most prevalent *Babesia* species reported in humans from different parts of the globe include *B. bigemina*, *B. bovis*, *B. crassa-like*, *B. divergens*, *B. duncani*, *B. microti*, *B. odocoilei*, and *B. venatorum* [292]. These *Babesia* spp. have the highest prevalence in Europe, lower middle-income countries, and among individuals with *a* history of tick *bites* and other tick-borne diseases [292].

Patients with active *Babesia* oftentimes required multiple rotations of antimalarial therapies, including clindamycin [293], atovaquone and azithromycin with or without sulfamethoxazole/trimethoprim [147], atovaquone/proguanil (Malarone) [149], tafenoquine [294], with or without ivermectin [151], lumefantrine/artemether (Coartem) [152], and botanicals (artemisinin, *cryptolepis sanguinolenta*, *Scutellaria baicalensis* (Chinese skullcap), *Polygonum cuspidatum* (Japanese knotweed) [156], as well as occasionally low dose mefloquine [153]. Oftentimes the treatments decreased symptoms and clinically appeared to lower the parasitic load (less fevers, sweats, chills, flushing, air hunger, cough); however, symptoms oftentimes relapsed when treatment was stopped. Newer and more effective therapies for babesiosis are therefore essential for the CLD population. Clofazimine might have some efficacy in resistant Babesiosis [295,296]; however, it is expensive, not commercially available in the US [297], and requires submission of a single-patient Investigational New Drug request to the FDA [298]. Clofazimine is also only effective in producing a radical cure of babesiosis when the infectious isolate belongs to the Munich strain of *B. microti*, which has been implicated in a few cases of human babesiosis, all of them found in Europe [299,300]. The only potential indication for mixing atovaquone and clofazimine instead of azithromycin is in the immunocompromised patient, as that combination has greater efficacy against a relapse with *B. microti* [297]. Many of our patients had evidence of immune suppression, making it more difficult to cure babesiosis [301,302,303].

*Babesia microti* has become resistant to standard therapies (atovaquone, azithromycin, clindamycin, and quinine) [304]. As per the 2018 Report of the HHS Other Tick-Borne Diseases and Co-Infections Subcommittee [305], the pathophysiology of Babesiosis includes potentially adverse effects on the treatment of Lyme disease. Effective control of *B. burgdorferi* infection depends on a Th2 CD4+ T cell response within regional lymph nodes, and co-infection with *B. microti* may influence T cells toward a TH1 response [305]. *Babesia* spp. may also complicate the course of DDDCT and HDDCT, as clinically they can result in hemolytic anemia [306,307], similar to tafenoquine [308] used to treat resistant and relapsing *Babesia* infections [150,294]. Tafenoquine should therefore be avoided during dapsone therapy since one of the *medications*’ side effects includes elevated levels of methemoglobin [308], similar to dapsone, potentially complicating the course of dapsone and methylene blue. Since dapsone and MB have been associated in the peer-reviewed literature with mild hemolytic anemia [309], and *Babesia* (and tafenoquine as an anti-malarial treatment) may also cause hemolytic anemia, it is incumbent on the physician to determine whether one or several etiologies are present. Although mild hemolytic anemia is common following dapsone treatment, it rarely warrants *a* change of therapy, as increased hemolysis usually only occurs in patients with glucose-6-phosphate dehydrogenase deficiency [310,311]. Measuring haptoglobin levels, reticulocyte counts, elevated unconjugated bilirubin, and LDH can determine if hemolysis is present and to what degree [312], or if the anemia is primarily due to dapsone’s effect on folic acid metabolism [310]. In the majority of our patients, folic acid inhibition was the primary cause of anemia, and abnormal laboratory values reversed at the end of DDDCT and HDDCT as long as patients remained on high doses of folic acid.

Laboratory values were closely monitored during the first month on DDDCT (week 3), then weekly (weeks 5–8) during the second month on DDDCT, and biweekly during the last week of an HDDCT pulse. Despite changes in a number of laboratory values during the course of treatment, all patient values returned to normal at the end of treatment (except for patient 24, who had a history of low platelet levels in the past). Laboratory values with a CBC, CMP (electrolytes, liver, and kidney function), and methemoglobin levels were monitored before, during, and after DDDCT and HDDCT. There were temporary changes in white cell counts (4 episodes of leucopenia, 1 episode of leukocytosis), platelets (3 patients had mild thrombocytopenia), transient elevation in myelocytes and metamyelocytes (4, 5 patients), regular decreases in hemoglobin (Hb) and hematocrit (Hct), and expected rises in the mean corpuscular volume (MCV) while on dapsone due to the drug’s effect as a folic acid inhibitor, affecting bone marrow production [110]. There were also transient mild liver function abnormalities (5 patients with changes in AST, 7 patients with changes in ALT), with 12 patients having transient elevations in bilirubin [171]. Methemoglobin (MetHb) levels rose in most patients due to dapsone’s potent oxidative effects [172]. Among 23 patients where methemoglobin levels were regularly measured on DDDCT and HDDCT, 65.2% (15/23) had MetHb values less than 5%; 26% (6/23) had Methb values between 5 and 10%; and 8.6% (2/23) had MetHb between 11–20%, as long as patients took the maximum dose of methylene blue (300 mg PO BID) with other antioxidants (glutathione, Vit C, Vit E, NADH) [173]. See Protocols 1 and 2 and Table 1 for the full protocol. Most patients had no symptoms of methemoglobinemia when the level was less than 5%, minimal symptoms between 5 and 10% (slight blue hands and lips, some dyspnea), and the two patients who had levels greater than 10% (pt 7, 15) tolerated the transient elevations without difficulty [173]. All abnormal values returned to normal over time. Cimetidine was not regularly used in our protocols to lower methemoglobin [120]; however, potentially could be added to the DDDCT and HDDCT protocols in patients who have MetHb levels greater than 10% or who are symptomatic at MetHb levels less than 10%, despite using higher doses of methylene blue and antioxidants (e.g., glutathione, NAC, NADH, Vitamin C, and Vitamin E). The N-hydroxylation of dapsone is thought to be responsible for causing methemoglobinemia and hemolysis associated with this drug, and concurrent cimetidine therapy might reduce some of the hematological side effects of dapsone [121]. This should be examined in future protocols using HDDCT for Lyme and *Bartonella* to further improve tolerability, as well as in other autoimmune dermatological diseases such as dermatitis herpetiformis, where dapsone is used in doses up to 400 mg per day [313,314,315]. A drug interaction checker should be used when using cimetidine and rifampin with DDDCT and HDDCT, as cimetidine can increase levels of certain medications (hydroxychloroquine) and decrease levels of certain medications (thyroid hormones), similar to rifampin’s effect of decreasing levels of certain medications (hydroxychloroquine, thyroid, and adrenal hormones) [316,317].

Liver functions were closely monitored on rifampin and PZA due to the potential of both medications to cause transaminitis [318,319]. AST levels were normal in 19/24 patients measured (79.2%); however, temporary borderline increases in AST were seen in 5 patients (mean increase 9.4 IU/L; median 9; range 9, minimum 4-maximum 13; SD = 3.51), and temporary mild increases in ALT levels were seen in 29.2% of patients (7/24; mean increase 24.86 IU/L; median 26; range 51; minimum 2, maximum 53; SD = 19.44). Transaminases returned to WNL post-dapsone combination therapy, as did total bilirubin levels, which were temporarily elevated. Alkaline phosphatase (ALP) levels were normal in 88% of patients, and 12% (3/24) had low alkaline phosphatase (patients 5, 9, and 10) before or during dapsone combination therapy. All 3 patients were in remission at the end of therapy and had a prior history of low B12 levels, which can be associated with low alkaline phosphatase [191]. A low alkaline phosphatase level may therefore be a surrogate marker of occult B12 deficiency [187], similar to elevated levels of methylmalonic acid [320]. Persistently low serum ALP may also be secondary to malnutrition, bisphosphonates, other vitamin and mineral deficiencies (zinc), hypophosphatasia, and endocrine disorders such as hypothyroidism [321]. A full diagnostic work-up should therefore be performed to determine if one or more causes of low ALP are present.

We regularly used liver support with NAC, ALA, glutathione, and sulforaphane [322], and added milk thistle when LFTs were elevated [323]. In some patients, NASH was present, resulting in low-level chronic elevations of transaminases [324]. Overall, dapsone combination therapy was found to be safe and effective in the treatment of CLD and associated co-infections, including *Bartonella*, when the protocols were adhered to, including the laboratory testing schedule. Higher doses of methylene blue and anti-oxidants, as well as folic acid supplementation, used in this HDDCT protocol compared to prior published higher dose dapsone protocols [102,103], may have contributed to its benefits, safety, and tolerability.

## 5. Conclusions

This is, to our knowledge, the first effective, short-term oral, generic protocol for treating CLD/PTLDS and associated co-infections, including *Bartonella*. We found that 8 weeks of DDDCT followed by a 4-day pulse of HDDCT was sufficient to put patients with CLD/PTLDS into long-term remission if they did not have evidence of active co-infections, i.e., *Babesia* and *Bartonella*. Patients who were co-infected with *Bartonella* were sicker, with more neuropsychiatric symptoms, immune deficiency, and increased autonomic and peripheral neuropathy. They required 8 weeks of DDDCT followed by a 6–7-day pulse of HDDDCT to improve and/or go into full remission. The 4-day HDDCT, although helpful in relieving symptoms in those with CLD and *Bartonella*, was insufficient to keep most co-infected patients with *Bartonella* in remission.

Patients with evidence of chronic *Bartonella* had superior results using 8 weeks of DDDCT followed by a 6–7-day pulse of HDDCT. This confirms the culture results from Johns Hopkins researchers looking at *Bartonella* persisters in culture, where 6 days of combination antibiotic therapy (rifampin, azithromycin, and methylene blue) were required to completely eliminate *Bartonella* persisters in biofilms [101]. We did not have a 100% success rate using 6 days of Plaquenil, a tetracycline (doxycycline or minocycline), rifampin, azithromycin, pyrazinamide, and dapsone with a higher dose of methylene blue; however, we had superior response rates in the general improvement of symptoms (100%) compared to past dapsone protocols [51,102,103,104,105]. Earlier protocols used 8 weeks of DDDCT with shorter courses of high-dose dapsone (4-day maximum) and lower doses of methylene blue. Higher methylene blue dosage as well as extending the length of time on high-dose dapsone (200 mg PO BID) from 4 days to 6–7 days improved response and remission rates, especially in those co-infected with *Bartonella*. Pyrazinamide may also have been helpful in inducing remission rates for *Bartonella*; however, larger controlled studies will be necessary to determine its beneficial effect [325,326]. Of 23 patients who completed a full 8-week course of DDDCT prior to one or several courses of HDDCT, 7/23 (30.5%) had a full resolution of their Lyme and tick-borne symptoms for 3 months or longer, even if there was prior evidence of co-infections, including *Babesia* and/or *Bartonella.* Three patients (13%) also had a full resolution of their Lyme and tick-borne symptoms for 1 ½ months or longer, having recently completed the full protocol 6 weeks prior, and age, length of illness, or gender did not affect remission rates or clinical improvement. Sixteen patients (64%) were sick for 10–20 years or longer, and 8/16 (50%) were in remission, with 8/16 (50%) showing significant clinical improvement.

We recognize that the tick-borne protocol presented here is complex, has potential gastrointestinal issues (nausea and vomiting) and hematological side effects with HDDCT, and requires a highly motivated health provider and a highly engaged patient. It is, however, a protocol that we have demonstrated to be safe and effective when patients follow the guidelines outlined in Table 1, Table 2 and Table 3 and Protocols 1 and 2. Our study brings hope to the Lyme community since this short-term, oral, generic antibiotic protocol can greatly improve and/or completely resolve symptoms in those who are ill, even for decades.

Climate change and the rise in global temperatures are one of the greatest threats to mankind’s health and are increasing our risk of vector-borne illness, whether it be mosquito-borne (West Nile, malaria) [327], flea-borne (*Bartonella*) [10] or tick-borne [328]. Lyme disease, known as a “great imitator”, is spreading in epidemic proportion, with a reported estimated global Bb seroprevalence of 14.5% [2], and climate change is expected to increase the number of LD cases in the United States by over 20% in the next several decades [329]. Borreliosis can cause numerous disabling rheumatologic, cardiologic, neurologic, and psychiatric complications, mimicking a broad range of chronic illnesses with constitutional symptoms, including chronic fatigue syndrome/M.E. [330], fibromyalgia [331], neuropsychiatric as well as autoimmune diseases [42,66,67]. The quality of life of those living with CLD/PTLDS has been reported to be worse than that of those living with congestive heart failure, diabetes, and rheumatoid arthritis [190,332], secondary to infection, inflammation, and immune dysfunction resulting in disabling symptoms [42]. Overlapping causes of inflammation on the 16-point MSIDS model found in our cohort of 25 patients with CLD/PTLDS that were responsible for their chronic illness included multiple chronic infections (*Borrelia*, *Babesia*, and *Bartonella*), dysbiosis, leaky gut with food sensitivities or mast cell activation syndrome, environmental toxins (mold, heavy metals), mineral deficiencies, and chronic insomnia. These six factors contributed to chronic inflammation, resulting in downstream effects including mitochondrial dysfunction, POTS/dysautonomia, liver dysfunction, autoimmunity, and hormonal dysregulation. Despite these overlapping variables, 100% of our patients had improvement in their tick-borne symptoms using this short-term, oral, generic antibiotic protocol, implying a major role for the biofilm/persister forms of the bacteria, i.e., *Borrelia* and *Bartonella*, in driving chronic symptomatology.

It was ultimately chronic *Babesia* spp. that played a major role in interfering with the success of DDDCT and HDDCT, and if questions 1 and 22 on the HMQ Lyme questionnaire are positive [194], it should alert the clinician to the possible presence of Babesiosis. None of the six patients who were *Babesia* FISH-positive remained in long-term remission. It is essential that we invest more research funding into finding effective treatments for chronic babesia. Patients who are immunosuppressed, splenectomized, and/or elderly with chronic health conditions, including cardiac and pulmonary problems, are at greater risk for lethal complications from *Babesia* [333]. Since *Babesia microti* is now resistant to standard anti-malarial therapies, with new species on the horizon, we need new tools and arsenals to fight these chronic parasitic infections, which are making chronic LD patients sicker. Investing in regular surveillance of ticks is also essential to determine the constantly changing environment of bacteria, viruses, and parasites [334]. Our paper describes some of the first cases of *Babesia odocoilei* being reported in the United States, and we lack sufficient knowledge regarding its spread among ticks in the Northern Hemisphere, the parasite’s pathogenicity, and/or how new emerging *Babesia* species may interact with the same host, causing illness. Similarly, several of our patients had simultaneous active co-infections with Lyme disease, different *Babesia* species, and different *Bartonella* species. We lack sufficient knowledge about the complex interactions of these organisms, and we suggest more research funding should also be invested in these areas.

Based on our patient population, anyone suffering from severe neuropsychiatric symptoms who also has significant physical complaints seen in CLD/PTLDS, i.e., chronic fatigue, headaches, migratory muscle/joint/nerve pain, cognitive difficulties, and a sleep disorder, should alert the clinician to the possibility of *Bartonella* exacerbating psychiatric symptoms. If the patient presents with classic *Bartonella* striae (see Figure 3) with or without granulomas on the extensor surfaces (see Figure 2), pain on the bottom of the feet, significant neuropathic symptoms, and/or an immunodeficiency, *Bartonella* should also be suspected as a potential underlying cause of illness. Due to the rising number of pathogenic *Bartonella* species, testing should include evaluation of multiple species by antibody, PCR, ddPCR (Galaxy Diagnostics, Research Triangle Park, North Carolina, USA), *Bartonella* FISH, and a *Bartonella* Immunoblot (IgeneX Laboratories) and/or T Labs (Gaithersburg, MD, USA). VEGF is also a useful surrogate marker for active *Bartonella* and can be obtained through local laboratories.

If a patient with CLD/PTLDS has failed multiple antibiotic protocols and has not yet tried a biofilm/persister protocol such as dapsone combination therapy, it is the first therapy that we would recommend based on our clinical experience, as long as there are no clear contraindications, i.e., G6PD deficiency, iron deficiency anemia, severe sulfa allergy, etc. Although the role of biofilm and persister bacteria in Borreliosis and Bartonellosis has been debated in the scientific literature, multiple publications on dapsone combination therapy have shown significant efficacy rates with short-term antibiotic therapies in the treatment of CLD/PTLDS using combination therapies with a persister drug, dapsone, along with biofilm agents. The chronic Lyme community has been desperate to find a cure for this chronic illness [335]. The “bench to bedside” approach used in our DDDCT and HDDCT studies demonstrates that answers are available, in large part due to the excellent culture-based research on *Borrelia* and *Bartonella* conducted at major universities [75,95,101,108,336,337] which guided our therapies. The next step needed to confirm these results is an 8–9-week double-blind, randomized, controlled trial of DDDCT followed by HDDCT for 4 days for the treatment of CLD/PTLDS without co-infections, based on our findings, or an 8–9-week RCT of DDDCT followed by a 6–7-day pulse of HDDCT for CLD with associated co-infections such as *Bartonella*.

Multiple infections, including *Babesia* and *Bartonella*, biofilm/persister medications, as well as MSIDS factors, were not regularly accounted for in prior randomized controlled trials for CLD/PTLDS evaluating the efficacy of chronic antibiotic therapy [338,339,340,341,342]. In our patient population, comprehensively addressing co-infections, using a biofilm/persister drug regimen with dapsone and methylene blue, and diagnosing and treating any MSIDS abnormalities were essential to patients recovering their health. Based on similar chronic fatiguing, musculoskeletal, and neuropsychiatric illnesses, including long COVID, where 7/16 abnormalities on the 16-point MSIDS model have been published in the scientific literature as being associated with long COVID, we believe that a paradigm shift from a one cause/one disease model is warranted when evaluating resistant chronic disease. Lyme disease and long-term COVID are now affecting tens of millions of individuals worldwide [2,215]. A precision medicine approach, screening for each of the 16 factors on the MSIDS model, may help determine the underlying etiologies of these chronic fatiguing illnesses, leading to more effective therapies and helping to lower rising healthcare costs while decreasing suffering and improving patient outcomes.

## Data Availability

All the data for this study are in the paper. There are no public archives containing information.

## Supplementary Materials

The following supporting information can be downloaded at: https://www.mdpi.com/article/10.3390/microorganisms11092301/s1.
