# Supplementary material for: Comparison of the Efficacy of Longer versus Shorter Pulsed High Dose Dapsone Combination Therapy in the Treatment of Chronic Lyme Disease/Post Treatment Lyme Disease Syndrome with Bartonellosis and Associated Coinfections
Source: Microorganisms. 2023 Sep 12;11(9):2301. doi: 10.3390/microorganisms11092301 (PMC10537894; doi:10.3390/microorganisms11092301)
Supplement: Supplementary file 1 [file microorganisms-11-02301-s001.zip › microorganisms-2562477-supplementary.pdf]

Supplement S1:

Methylene Blue Consent

Patient Name:____ DOB:____________ Date:_____/_____/_____

Methylene blue is used for the treatment of idiopathic and drug-induced methemoglobinemia. It is also being used to help prevent methemoglobinemia while taking the Dapsone treatment protocol. Methylene blue has also been shown to have efficacy as a 'persister' drug for Lyme disease and Bartonella, as per Johns Hopkins researchers.

The drug will not reverse methemoglobinemia in patients with glucose-6-phosphate dehydrogenase (G-6-PD) deficiency and may induce an acute hemolytic episode in these patients, which is why we require this lab test to be checked prior to you starting Methylene blue (American Society of Health System Pharmacists. AHFS Drug Information 2008. Bethesda, Maryland 2008 URL: https://toxnet.nlm.nih.gov/cgi-bin/sis/search/r?dbs+hsdb:@term+@rn+@rel+61-73-4). Long-term administration of methylene blue may result in marked anemia due to accelerated destruction of erythrocytes; hemoglobin concentrations should be checked frequently.

Methylene blue cannot be taken with:

Contraindicated
5-HTP
amitriptyline
amoxapine
amphetamine
bupropion
buspirone
citalopram
clomipramine
codeine
desipramine
desvenlafaxine
deutetrabenazine
dextroamphetamine
dihydrocodeine
doxepin
duloxetine
escitalopram
fluoxetine
fluvoxamine
imipramine
isocarboxazid
levomilnacipran
lisdexamfetamine
meperidine
methamphetamine
milnacipran
mirtazapine
morphine
nefazodone
nortriptyline
paroxetine
phenelzine
procarbazine
protriptyline
ropinirole
safinamide
selegiline transdermal
sertraline
tapentadol
tramadol
tranylcypromine
trazodone
trimipramine
venlafaxine
vilazodone
vortioxetine

Avoid/Use Alternative
alfentanil
buprenorphine
butorphanol
fentanyl
hydrocodone
hydromorphone
levorphanol
linezolid
lorcaserin
methadone
nalbuphine
oxycodone
oxymorphone
pentazocine
remifentanil
rizatriptan
sufentanil
sumatriptan
zolmitriptan

Caution Advised
almotriptan
bromocriptine
cabergoline
carbamazepine
cocaine topical
cyclobenzaprine
dextromethorphan
dihydroergotamine
dolasetron
eletriptan
ergotamine
frovatriptan
granisetron
isoniazid
methoxsalen
methoxsalen topical
methylergonovine
naratriptan
ondansetron
oxcarbazepine
palonosetron
rasagiline
selegiline
St. John's wort
yohimbe
yohimbine

If any of the following side effects occur, please contact our office immediately:

Serious Reactions

- hypersensitivity rxn
- anaphylaxis
- hemolytic anemia
- serotonin syndrome
- syncope

Common Reactions

- extremity pain
- urine discoloration
- fecal discoloration
- hot flashes
- dizziness
- hyperhidrosis
- skin discoloration
- nausea

- headache
- syncope
- chest pain
- diaphoresis
- vomiting
- back pain
- abdominal pain
- photosensitivity

I understand that I will need to have my labs checked every week (CBC, CMP, methemoglobin levels) as the Dapsone protocol medications may cause significant anemia and raise methemoglobin levels (resulting in potentially serious side effects including increased fatigue, headaches, and shortness of breath, rarely requiring IV medication in an emergency room setting). To minimize that effect, and reduce methemoglobin levels, I understand the need to avoid any other off label treatments during this time (including ozone and oxidative therapies) that have not been discussed with my provider, along with the need to take a minimum of certain antioxidants including: 1000 mg of liposomal glutathione twice a day (Essential Pro, 4 capsules, twice a day) along with high dose folic acid. This would include using high dose Leucovorin (25 mg, 2-4 tablets twice a day combined with 5 MTHF-ES, 4 pills twice a day, or Folafy-ER (Xymogen, 15 mg of L-methyl folate) as folic acid support. Other antioxidants used to help lower methemoglobin include Vitamin C (1-2 grams twice a day), Vitamin E (300 IUs twice a day), NADH (ENADA) 5 mg twice a day. In certain cases of elevated methemoglobin despite using methylene blue and the above antioxidants, cimetidine 400 mg twice a day may be added to your regimen. In that case, your provider will review potential medication interactions, and discuss lowering hydroxychloroquine (Plaquenil) to once a day.

Please verify that our office has received your lab results by sending a weekly email to our office (medical@hvhac.com) letting us know the day and time you went to the lab so that we can follow up with the results. If you have any questions before, during, or after the study, please contact our office at medical@hvhac.com

For emergencies, please call the office number, 845-229-8977, and you will be given instructions on how to reach the on-call medical provider. For any serious side effects, go to your local emergency room.

It is essential that while on Dapsone, you must be on several folic acid supplements to help decrease the possibility of severe anemia (i.e. Leucovorin 25mg twice a day and 5 MTHF-ES, 4 pills twice a day or Folafy ER, 2-4 pills twice a day). If you have a history of being sensitive to high doses of methylation support which may cause symptoms such as irritability and/or agitation, please inform your provider. In rare cases you could also experience nerve pain (neuropathy- although most patients in our study showed statistical improvement of neuropathy on Dapsone), and you should inform the office promptly if you have any unusual symptoms while on this protocol.
You must also use NAC 600 mg twice a day, alpha lipoic acid twice a day (i.e., ALAMAX 600 mg) along with liposomal glutathione (i.e., Essential Pro), 4 capsules (500 mg) twice a day to support detoxification pathways and help minimize the possibility of methemoglobinemia (NAC and alpha lipoic acid helps to increase glutathione levels, and glutathione has been shown to be helpful in reducing methemoglobinemia).
For biofilms, speak to your provider about which biofilm agents will be used during dapsone combination therapy. This may include Stevia 15 drops twice a day, along with cinnamon/clove/oregano oil capsules twice a day (Doctors Best) or oregano oil capsules 60 mg) twice a day; along with Biocidin, two sprays twice a day, and peppermint oil capsules twice a day. For cystic forms of borrelia, as well as Plaquenil (hydroxychloroquine) 200 mg twice a day, you may use grapefruit seed extract, two twice a day (Pure Encapsulations). Nystatin tablets, 500,000 Units two twice a day with a sugar free diet is also important to reduce the possibility of yeast overgrowth in the gastrointestinal tract.

At the end of the protocol, you will be stopping all medication used to treat your tick-borne disorders except for your folic acid (Leucovorin and 5 MTHF ES or Folafy-ER) and probiotics, as well as your biofilm agents. Please speak to your healthcare provider about using a one month mitochondrial regeneration protocol with any other specific instructions for your care once you are done with the dapsone protocol. Please have blood work repeated at the laboratory one week and then 3-4 weeks off the protocol. Stay on all other supplements and medications prescribed by your healthcare provider after you are done with the Dapsone, unless specifically indicated.

In signing this document, I understand the necessity of regular laboratory testing listed above, as well as strict compliance with all dietary and supplement regimens prescribed by my medical providers. I will avoid all alcohol and adhere to a strict yeast free, and sugar free diet as instructed by my provider. I also understand I can stop or adjust the protocol at any point in time if needed, by notifying Dr Horowitz and his medical staff at medical@hvhac.com.

Patient Signature:____Date:__________________

Witness Signature:
____Date:__________________

Start date: _____________________Expected end date: ________________
